# Measuring single constructs by single items: Constructing an even shorter version of the “Short Five” personality inventory

**DOI:** 10.1371/journal.pone.0182714

**Published:** 2017-08-11

**Authors:** Kenn Konstabel, Jan-Erik Lönnqvist, Sointu Leikas, Regina García Velázquez, Hiaying Qin, Markku Verkasalo, Gari Walkowitz

**Affiliations:** 1 Department of Chronic Diseases, National Institute for Health Development, Hiiu, Tallinn, Estonia; 2 School of Natural Sciences and Health, Tallinn University, Tallinn, Estonia; 3 Institute of Psychology, University of Tartu, Tartu, Estonia; 4 Swedish School of Social Science, University of Helsinki, Finland; 5 Institute of Behavioural Sciences, Department of Psychology and Logopedics, University of Helsinki, Finland; 6 Nankai Institute of Economics, School of Economics, Nankai University, P.R. China; 7 University of Cologne, Department of Corporate Development and Business Ethics, Albertus-Magnus-Platz, Cologne, Germany; Universitat Wien, AUSTRIA

## Abstract

The aim of this study was to construct a short, 30-item personality questionnaire that would be, in terms of content and meaning of the scores, as comparable as possible with longer, well-established inventories such as NEO PI-R and its clones. To do this, we shortened the formerly constructed 60-item “Short Five” (S5) by half so that each subscale would be represented by a single item. We compared all possibilities of selecting 30 items (preserving balanced keying within each domain of the five-factor model) in terms of correlations with well-established scales, self-peer correlations, and clarity of meaning, and selected an optimal combination for each domain. The resulting shortened questionnaire, XS5, was compared to the original S5 using data from student samples in 6 different countries (Estonia, Finland, UK, Germany, Spain, and China), and a representative Finnish sample. The correlations between XS5 domain scales and their longer counterparts from well-established scales ranged from 0.74 to 0.84; the difference from the equivalent correlations for full version of S5 or from meta-analytic short-term dependability coefficients of NEO PI-R was not large. In terms of prediction of external criteria (emotional experience and self-reported behaviours), there were no important differences between XS5, S5, and the longer well-established scales. Controlling for acquiescence did not improve the prediction of criteria, self-peer correlations, or correlations with longer scales, but it did improve internal reliability and, in some analyses, comparability of the principal component structure. XS5 can be recommended as an economic measure of the five-factor model of personality at the level of domain scales; it has reasonable psychometric properties, fair correlations with longer well-established scales, and it can predict emotional experience and self-reported behaviours no worse than S5. When subscales are essential, we would still recommend using the full version of S5.

## Introduction

### The “Short Five” (S5) personality questionnaire

The “Short Five” (S5 [1]) is a personality inventory based on the Five-Factor model (FFM [2]) that was designed using a principle of comprehensive single items: each item was written to capture either the low or high end of a predefined lower-order trait dimension. The lower-order structure of the S5 is based on the subscales (“facets”) of the NEO PI-R [3] as one of the most widespread ways to conceptualize the FFM. Despite being significantly shorter, the S5 has high correlations with its longer counterparts, has similar psychometric properties, and is no worse in predicting emotional experience and various self-reported behaviours as external criteria [1].

Construction of the 60-item S5 was partly motivated by the need to save respondents’ time while not giving up the possibility of measuring lower-order traits—other comparable measures of the FFM often have four (NEO PI-R [3]) of five (EPIP-NEO [4]) times this number of items. However, even 60 items may be too much [5], especially in large survey studies where personality is only one among several characteristics to be measured. There are several shorter, reasonably good 10 to 30-item questionnaires based on the FFM or “Big Five” [5–6] but the comparability (meaning equivalence) of their scores to those of the longer, well-established questionnaires, can be questionable. One reason for this is that the scales of short questionnaires often consist of a few prototypical items that may represent what the researchers or respondents believe to be the “core” of a trait, but do not systematically cover all aspects of it. The possibility of systematic coverage of a broad trait such as extraversion may even be believed impossible to achieve with, say, 6 items, let alone a single item. Another reason is that a typical item in a short questionnaire is itself short, often consisting of one or two adjectives. Such items may evoke hasty, unreflective answering, are difficult to translate from one language to another (as the exact meaning nuances of personality descriptive adjectives can seldom be translated exactly), and may often be understood in idiosyncratic ways [1]. There is hence a clear need of short personality questionnaires which would maintain comparability with longer questionnaires as much as possible. A 30-item version of S5 using just positive indicator of each subscale has been used in the past [1,7] but is unlikely to be the optimal solution because of the problem of acquiescence, and because the keying of traits is arbitrary (a person may be equivalently described as scoring high on optimism, or low in pessimism, presuming that the two are opposite) and so for some traits, the “negative” indicator may in fact be more informative.

### The problem of acquiescence

According to the good practice, personality scales should be balanced, that is, half of the items should be worded so that higher agreement implies higher standing on the trait, and the other half, the other way round. This balanced keying is intended to cancel out the effects of acquiescent responding—in other words, indiscriminate agreement irrespective of the item content [8].

It is too much to hope that balanced keying would completely remove any variance related to acquiescence. This would need several improbable assumptions: for example, positively and negatively keyed items should, on the average, be equal on acquiescence, and acquiescent responding should work in essentially the same ways regardless of whether the item contains negation (which is common in reverse keyed items.) However, balanced keying is also necessary to be able to separate content from acquiescence, by providing a rough method of measuring indiscriminate agreement regardless of item content [9–10].

McCrae, Herbst and Costa [8], showed that controlling for acquiescence in TCI and MPQ resulted in better conformity to the five-factor model. Some recent results suggest that controlling for acquiescence can, at least in representative samples, improve the psychometric properties of personality measures [11–13]. Balancing is obviously not possible with single-item scales, and one needs to rely on other ways of controlling acquiescence. And likewise, because one cannot rely on acquiescence being at least partly “washed out” by balanced keying as in multi-item scales, it is an especially important topic to study in single-item scales.

In a balanced scale (i.e. having an equal number of positive and negative indicators), acquiescence can be approximated by the sum (or mean) of all untransformed items. If acquiescence is not an issue, then a person would answer opposite items (e.g. “I am a highly talkative person” vs “I am quiet and reserved”) in opposite direction, so the sum of untransformed items would be low. If a person tends to agree indiscriminately with items, then the sum of untransformed items would be high; at the same time, their score on the content scale would be close to the scale midpoint.

Acquiescence can be controlled for by partialling it out from the scales [8], or, as recommended by Rammstedt and colleagues [13], by subtracting the mean acquiescence score from all items.

### Aims of the present study

The present study had, thus, the following aims.

1. To shorten the S5 so that each subscale would only have a single item. More specifically, we intend to find an optimal subset of 30 items in such a way that the five domain scales would still be balanced, having an equal number of positive and negative indicators.
2. To compare the 30-item “Extra Short Five” (XS5) to the unabridged S5, in terms of prediction of external criteria, as well as psychometric properties traditionally used to evaluate personality questionnaires (internal reliability, principal component structure).
3. To investigate whether different ways of controlling for acquiescence can be recommended for use with XS5; whether they result in better prediction of criteria, and / or better psychometric properties (a sign of meaning equivalence).

In constructing the S5, we relied on the idea of “Comprehensive Single Items” (CSI [1]) where each item is written to match, as closely as possible, the consensual expert definition of the trait it is intended to measure. For the present purpose, we have, essentially, to select one item from among two for each subscale, with the additional constraint of achieving a balanced keying at the domain level. As there is no single best criterion of which item is better, we used a combination of several criteria:

1. Correlation with longer personality questionnaires (NEO PI-R and EPIP);
2. Self-peer correlations;
3. Internal homogeneity of the items. Respondents have occasionally complained about a few S5 items consisting of “several different parts” to which they would like to respond differently. To some extent, this is an inevitable by-product of the CSI approach used in constructing the S5. However, for the short version of the scale, we decided to avoid such “multi-barreledness” (two or more different questions being presented as a single item) as much as possible.

In Study 1, the purpose was to shorten the 60 item S5 into the new 30 item XS5. This was done by re-analysing the using the same university student data that was used to validate the original S5 [1]. The psychometric and predictive properties of the XS5 were investigated both when controlling for acquiescence responding and when not controlling for it. We examined the predictive properties of the XS5 using as criterion variables longer personality measures, peer-reports of personality, emotional experiences, and various behaviours.

A short measure such as the XS5 is most likely to be useful in large-scale studies conducted with other populations than university students. Therefore, in Study 2, we investigated the psychometric properties of the XS5 in a large representative sample from Finland. Higher levels of acquiescent responding can be one of the reasons that the psychometric properties of personality measures tend to be worse in representative samples as compared to student samples [11–13]. We therefore expected the psychometric properties of the XS5 to improve when controlling for acquiescence in Study 2. We also investigated whether controlling for acquiescence impacted the psychometric and predictive properties of the XS5 in the Study 1 student samples, but did not expect acquiescence to have much of an effect in those samples.

## Study 1

### Method

#### Participants

Much of the data that we employed in developing the XS5 was used in the development of the S5 [1]. The data set was constituted by Estonian (*N* = 393, 120 male, mean age 19.4, SD = 1.4), Finnish (*N* = 675, 338 male, mean age 24.5, SD = 5.4), English (*N* = 98, 31 male, mean age 20.9, SD = 3.5), and German (*N* = 216, 104 male, mean age 25.0, SD = 4.3) student samples (for some analyses, only a subset of the participants was available).

In addition, in order to make the S5 available in two of the most widespread languages of the world, new data were gathered in Spain and China. The Spanish sample was gathered at the University of Huelva and consisted of 257 students, with a mean age of 20.3 (SD = 5.8) years. First-year students anonymously completed the paper and pencil questionnaires as part of a class requirement for an introductory psychology course. The Chinese sample consisted of 192 students from various faculties, in all 122 women and 70 men with a mean age of 21.6 (*SD* = 2.8) years. The Chinese sample was gathered at the Nankai Institute of Economics. The study was advertised in the university newspaper and participants signed up via the institution’s website. The paper and pencil personality questionnaires were anonymously completed in group testing sessions. Participants' payments, which averaged 30.1 Yuan (*SD* = 12.8), were in part determined according to the decisions that they made in an unrelated decision-making task that was administered within the same testing session.

#### Multi-barreledness ratings and controlling for acquiescence

We also gathered some new data on the multi-barreledness of the S5 items. Ratings of multi-barreledness were obtained from 82 Finnish psychology students; they were asked to rate each item of the S5 according to how difficult they were to respond to. With “difficulty” we explicitly referred to such difficulty that was caused by two or more different questions being presented as a single item. See Table A in S1 File for the exact instructions and response options.

The psychometric and predictive properties of the XS5 were investigated both when acquiescence was and was not controlled for. For each participant, we computed, as an index of acquiescence responding, the mean of all items. There are two well-established ways to control for acquiescence responding. One way is to partial out the acquiescence score [14] in all analyses. Another way is to subtract the acquiescence score from each item. We used both ways in order to avoid method effects.

#### Construction of the XS5

In the S5, each of the 30 facets that constitute the FFM is measured by both a positively and negatively keyed item (adding up to twelve items per personality factor), and each item is responded to on a scale from -3 (*The description is completely wrong*) to 3 (*The description is completely right*). Personality facet and factor scores were obtained by first reverse coding the negatively keyed items, and then computing the mean. The items of the S5 are designed with an eye on comprehensiveness. For instance, the negatively keyed item that measures the Warmth facet of Extraversion is “I do not like to associate with people much; I am considered a rather cold and distant person”.

The XS5 was developed by selecting 30 items from the S5. Because there are two items (a positively keyed and a negatively keyed item) in each S5 subscale, the two constraints that we imposed amount to choosing either the “positive” or “negative” item to represent each subscale, with the restriction that there be exactly 3 “positively” keyed items in each domain. Within these constraints, the selection of items for the XS5 was based on the following three criteria:

1. Correlations with longer questionnaires. The correlations with NEO PI-R and EPIP-NEO in four countries [1] were z-transformed and averaged, weighted by the sample size in the respective countries.
2. Self-peer correlations and peer-peer correlations (Estonian sample [1]) were z-transformed and averaged (i.e., z-transformed self-peer correlations for each item were added to the z-transformed peer-peer correlations, and the result was divided by 2).
3. Ratings of multi-barreledness were obtained from 82 Finnish respondents; they were asked to rate each item of the S5 to the degree of difficulty in responding to it resulting from two or more different questions being presented as a single item.

Because there was no clear rationale to prefer one criterion over the others, all three criteria were standardized and summed to form a total score. The results of these analyses are presented in the supplementary online material (see Tables S2 and S3). The 30 items that were chosen for the XS5 based on the above three criteria are presented in the Appendix.

#### Other personality measures

The five factors and 30 facets of the FFM [3] were measured with the S5 [1], the 240 item Finnish [15], German [16], English [3], Spanish [17] and Chinese [18] versions of the NEO PI-R, and with the 300-item EPIP-NEO [6], a 240-item Estonian version of the IPIP-NEO [19–20] inventory that measures roughly the same traits as NEO PI-R.

#### Criterion variables

Regarding the criterion variables for our validity analyses, besides scores on the longer personality measures, we used peer-reports of personality and self-reports of emotional experiences and various behaviours. Peer-reports of personality were gathered in the Estonian sample by having each participant find two acquaintances who described them using a third-person version of the S5.

The behaviour report form (BRF [21]), administered to 116 of the German participants, is a self-report measure designed to assess several complex behaviours of some social, personal and even cultural significance, such as smoking and drinking behaviour, participation in sports, ability to play musical instruments, and so on. Our somewhat updated version of the BRF is presented in detail in our previous paper [1].

Emotional experience was measured in the Estonian sample using a five-point scale on which participants described how often they experienced 13 different emotions (fear, anger, sadness, joy, contempt, disgust and surprise, embarrassment, envy, shame, pride, guilt and jealousy).

### Results

We first evaluated the psychometric properties of the XS5. The internal consistency reliabilities averaged, across the four samples, .75, .74, .62, .51, and .65, for N, E, O, A, and C, respectively (see Table D in S1 File for a country level breakdown). These reliabilities improved somewhat and to similar degree when acquiescence was controlled for, either by partialling or by subtraction; applying the former method gave average reliabilities of .75, .76, .64, .53, and .68, respectively, whereas the latter method gave 75, .76, .66, .57, and .70, respectively (see Table D in S1 File for country-level breakdown).

After targeted Procrustes rotation towards the normative North-American NEO PI-R structure [3], the S5 factors showed fair to good congruence, with congruence coefficients averaging .90, .87, .84, .85, and .89, for N, E, O, A, and C, respectively (see Table E in S1 File). Regardless of which method was used to control for acquiescence, doing this tended not to affect the congruence coefficients, which were, for the method of partialling, .88, .86, .85, 86, and .90, respectively, and for the method of subtraction .88, .86, .83, .84, and .91. The matrix congruence coefficients were almost identical in the three cases: 0.87 in untransformed data, 0.87 after partialling for acquiescence, and 0.86 after subtracting acquiescence.

Because the principal component structures of S5 for the Spanish and Chinese samples have not been previously reported, these are shown in Tables P and R in S1 File, along with the corresponding XS5 structures (Tables O and Q in S1 File).

In addition to the principal component analysis, we conducted an exploratory structural equation model (ESEM) using data from all six countries with M-plus 6.12 and MLR as estimator. In a less restricted model, the 30 subscales of XS5 were allowed to have different factor loadings in each country. Orthogonal target rotation was used using binary target matrix. This model did not fit the data: χ^2^ (1770) = 3775.48, p < 0.0001; some but not all fit indexes were satisfactory: RMSEA = 0.061; CFI = 0.852; TLI = 0.781; SRMR = 0.041. In another, more restricted model, we constrained all factor loadings to be identical across 6 countries. This model did not fit the data either: χ^2^ (2395) = 4538.975; p < 0.0001; RMSEA = 0.058; CFI = 0.842; TLI = 0.827; SRMR = 0.061. Even though the fit indexes were not unanimously worse, the difference in χ^2^’s was significant as determined by the Satorra-Bentler equation [22]: χ^2^ (625) = 882, p < 0.0001.

We ran identical models with NEO PI-R and found, similarly, that neither models fit the data. Estonia was omitted from these models as a different questionnaire, EPIP-NEO was used there. (Omitting Estonian data from the corresponding models with XS5 would not have changed the conclusions.) For comparability with XS5, we used subscale scores rather than single items in both models. For the less restricted model, the fit statistics were: χ^2^ (1475) = 3038.23, p < 0.0001; RMSEA = 0.079; CFI = 0.858; TLI = 0.790; SRMR = 0.042. For the more restricted model, the fit statistics were: χ^2^ (1975) = 3649.463; RMSEA = 0.071; CFI = 0.848; TLI = 0.832; SRMR = 0.091. The difference was significant according to the Satorra-Bentler equation: χ^2^ (500) = 699, p < 0.0001.

As the invariance of factor loadings did not hold, we did not proceed to more restrictive levels of invariance. However, we investigated more closely two pairs of countries where one might expect more similarity: Estonia and Finland on the one hand, and Germany and UK on the other hand. In the Estonia vs Finland comparison, the fit statistics were: χ^2^(590) = 1601.95, p < 0.0001; RMSEA = 0.057; CFI = 0.878; TLI = 0.821; SRMR = 0.036 for the unrestricted model, and χ^2^(715) = 1803.74, p < 0.0001; RMSEA = 0.053; CFI = 0.869; TLI = 0.841; SRMR = 0.047. The difference was significant according to the Satorra-Bentler equation: χ^2^ (125) = 221, p < 0.0001. For the UK vs Germany comparison, the fit statistics were: χ^2^(590) = 1085.819, p < 0.0001; RMSEA = 0.073; CFI = 0.786; TLI = 0.685; SRMR = 0.051 (unrestricted model), and χ^2^(715) = 1149.112, p < 0.0001; RMSEA = 0.062; CFI = 0.813; TLI = 0.772; SRMR = 0.064. The difference was not significant according to the Satorra-Bentler equation: χ^2^ (125) = 110, p = 0.820. We did not thus find factorial invariance in the first comparison but did find it in the second.

We also fit the ESEM models in each country separately; the fit statistics are presented in Table S in S1 File. CFI varied from 0.769 (UK) to 0.883 (Estonia), and SRMR varied from 0.035 (Finland) to 0.056 (UK). Comparable results for the longer questionnaires (EPIP-NEO and NEO PI-R) are shown in Table T in S1 File.

**Fig 1:**
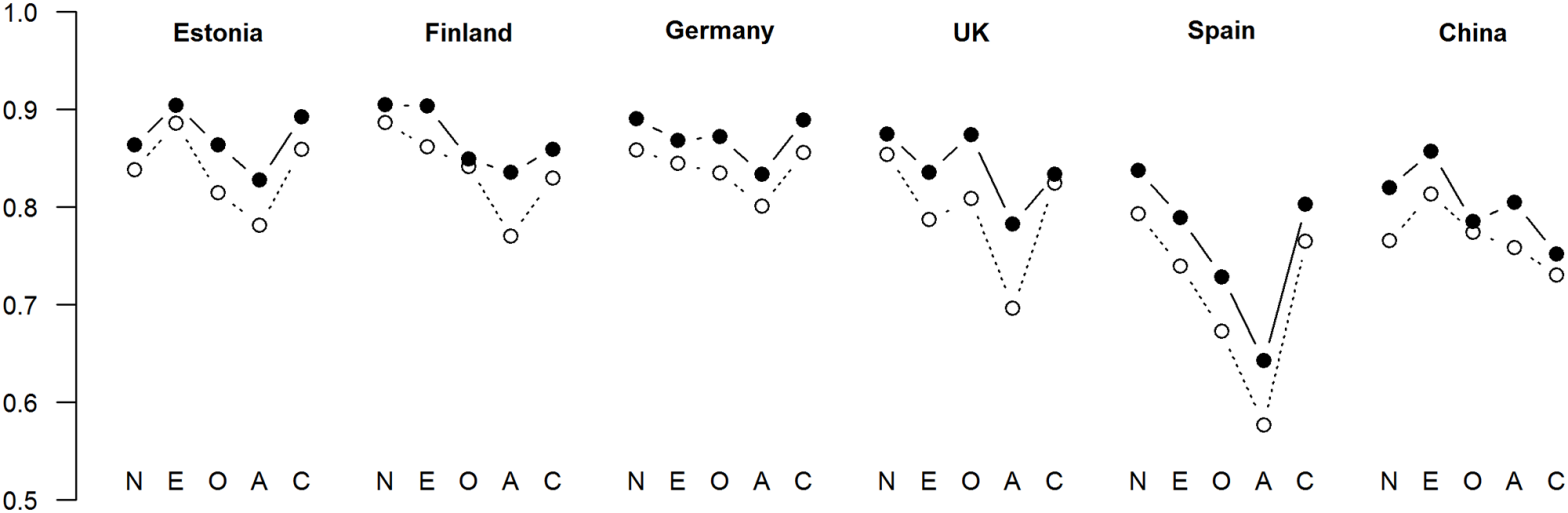
Correlations of the S5 and XS5 with longer personality inventories (EPIP-NEO in Estonia, NEO PI-R in other countries). Filled circles = S5; empty circles = XS5. Numeric data are presented in Tables F-K in S1 File.

We next computed the correlations between the XS5 and the EPIP-NEO (Estonian sample) or NEO PI-R (other samples). The domain-level correlations are shown in Fig 1. For comparison purposes, the correlations between the S5 and these longer measures are also plotted. The correlations between XS5 Agreeableness and the longer measures look somewhat weaker than the other cross-instrument correlations—this cannot be considered very surprising bearing in mind the modest internal consistency of the XS5 Agreeableness scale. Across culture, the correlations for this scale averaged .75, whereas all other scales showed average correlations between .79 and .84 (see Tables F-K in S1 File). The facet level correlations averaged, across the four countries and the thirty facets, .61 (Tables F-K in S1 File). Controlling for acquiescence slightly but consistently decreased the correlations between the XS5 and longer measures (see Tables F-K in S1 File).

**Fig 2:**
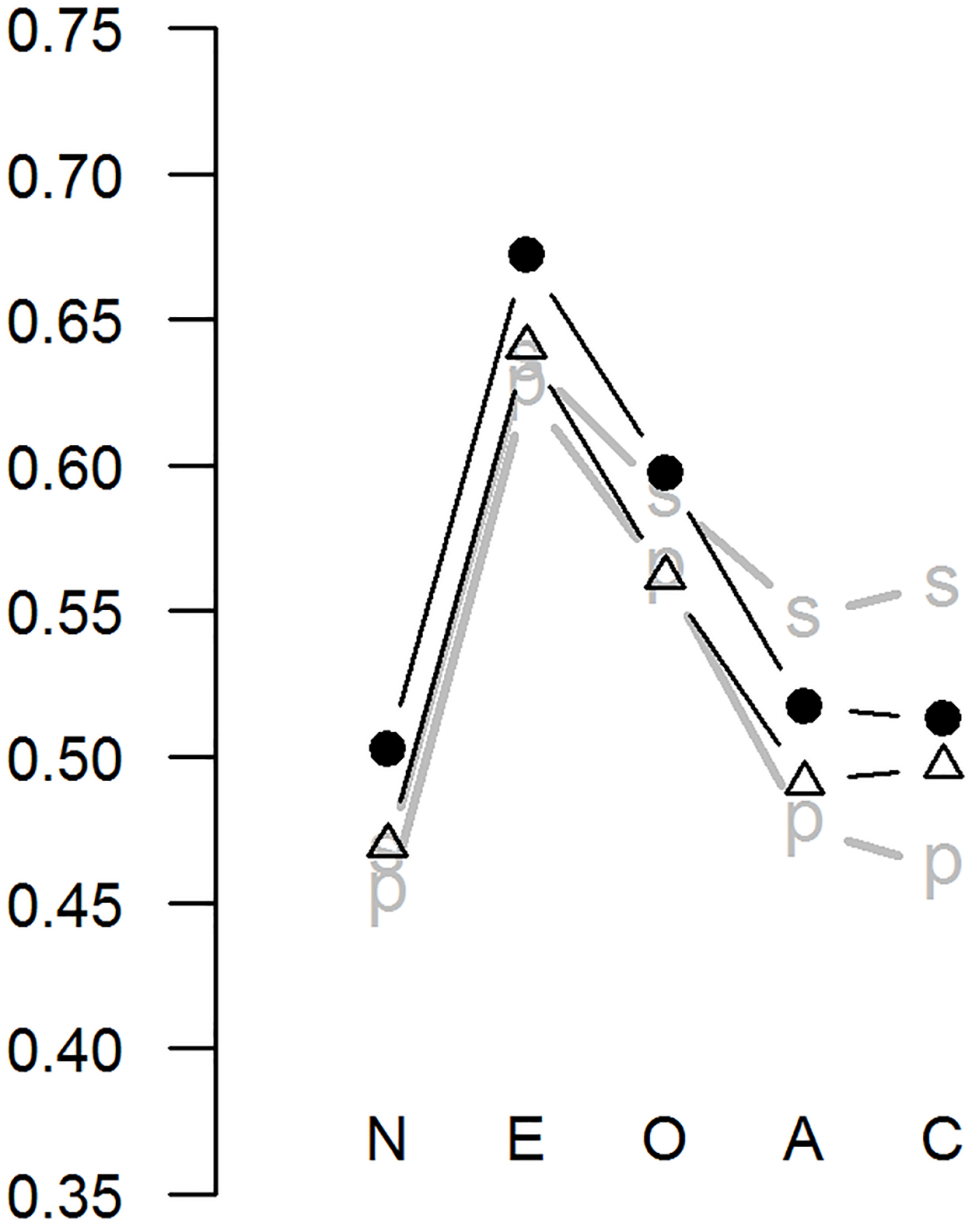
Self-peer correlations on five-factor domain scales: S5 (black circles) and XS5 (triangles). Gray lines indicate the correlations after controlling for acquiescence ("s" = subtracting, "p" = partialling). Numeric data are shown in Table L in S1 File.

To further assess the validity of the XS5 we computed self-peer correlations. The domain-level correlations are presented in Fig 2, along with the corresponding correlations for the S5 (see Table L in S1 File for facet level correlations). The self-peer correlations were very similar for these two measures. Controlling for acquiescence using the subtraction method improved these correlations somewhat (see Fig 2).

Our third criterion variable was emotional experience (this was available only in Estonian sample). We compared the XS5 and S5 scales in the prediction of self-ratings of emotional experience. Neuroticism and Extraversion should be the best predictors of emotional experience [23]. We constructed three linear regression models for each of the 13 dependent variables: the first including the XS5 Extraversion and Neuroticism scales as predictors, the second including the same S5, and a third model including both scales. Table 1 presents the variance explained in all three regressions. Note that XS5 had a slight advantage over S5 in predicting fear, anger, contempt, disgust, and guilt experiences. This may be counterintuitive but apparently, the items that were left out from XS5 contained no useful information for the prediction of these emotions and thus only confounded the true relationship (in some cases, as a reviewer suggested, there might have been a suppression effect). However, the differences were small. For guilt, the difference was 2.6% in the variance accounted for. On the other hand, S5 outperformed XS5 in predicting surprise, pride, and jealousy—the difference being the largest for jealousy (1.8% of the variance). Controlling acquiescence from the XS5 scored reduced R-squares (on the average, 0.0091with partialling, and 0.013 with mean subtraction).

**Table 1:** Predicting emotional experience from N and E: S5 contrasted with XS5 (Estonian sample, Study 1).

	S5	XS5	Both	Incr
Embarrassment	**0.22**	**0.21**	**0.22**	
Fear	**0.28**	**0.29**	**0.29**	
Anger	**0.15**	**0.16**	**0.17**	X
Envy	**0.11**	**0.11**	**0.12**	
Sadness	**0.31**	**0.30**	**0.31**	
Shame	**0.15**	**0.15**	**0.16**	
Joy	**0.18**	**0.18**	**0.19**	
Contempt	**0.03**	**0.04**	**0.05**	X
Disgust	**0.05**	**0.06**	**0.07**	X
Surprise	**0.03**	**0.03**	**0.04**	
Pride	**0.02**	**0.02**	**0.03**	S
Guilt	**0.14**	**0.16**	**0.17**	X
Jealousy	**0.13**	**0.11**	**0.13**	S

*Note*. R^2^-s for statistically significant models (p < 0.05) are shown in bold. Incr, incremental validity: ‘s’ means that S5 showed incremental validity over XS5; ‘x’ means that XS5 showed incremental validity over S5 (i.e. technically, that the model containing the corresponding scales from both questionnaires is statistically significantly better than the model containing only respectively XS5 or S5 as predictors).

Finally, we carried out a similar set of analyses in the German sample, comparing the XS5 and the S5 in terms of predicting participants’ behaviours. Separate linear models were constructed to predict each of the self-reported behavioural variables from the BRF, using all domains as predictor variables. As can be seen in Table 2, the S5 and XW5 were very evenly matched in the prediction of various behaviours. Controlling for acquiescence from the XS5 scores reduced R-squares (on the average, 0.00064 with partialling, and 0.0028 with mean subtraction).

**Table 2:** Predicting BRF criteria from S5 and XS5 domain scales (German sample, Study 1).

	S5	XS5	Both	Incr
Alcohol consumption	**0.281**	**0.274**	**0.308**	
Driving fast	**0.291**	**0.297**	**0.305**	
High school GPA	**0.223**	**0.219**	**0.313**	s,x
Self Enhancement	**0.163**	**0.168**	**0.226**	
Internet surfing for recreation	**0.175**	**0.170**	**0.208**	
BMI	**0.144**	**0.180**	**0.205**	
Internet surfing for work	0.118	0.115	0.160	
Dating frequency	**0.121**	0.107	0.140	
Traffic violations	0.093	0.062	0.164	
Routinely Exercises	0.106	0.118	0.139	
Participation in sports	**0.137**	**0.137**	0.169	
Tobacco consumption	**0.123**	**0.120**	0.153	
Fraternity interest	0.083	0.088	0.128	
Parties attended	0.103	0.091	0.116	
Last semester GPA	0.080	0.066	0.098	
Studies type	0.110	0.114	0.145	
Plays musical instrument	0.094	0.097	0.128	
Blood donations	0.066	0.107	0.176	x
Dating variety	0.075	0.082	0.100	
Dieting behaviour	0.109	0.115	0.177	
Internet chats with strangers	**0.205**	**0.201**	**0.209**	
Part time work	0.047	0.054	0.096	
Buys lottery tickets	**0.226**	**0.248**	**0.295**	
Preference for contacts	0.046	0.055	0.078	
Medication usage	0.086	0.088	0.177	

Note: R^2^ values for significant (p < 0.05) models are shown in bold. The column labelled ‘Incr’ refers to comparing the regression models with the domain scales of one questionnaire (NEO PI-R, S5 or NEO FFI) as predictors, with the model where both scales are used–that is incremental validity of one questionnaire over the corresponding scales of the other questionnaire. ‘x’ means incremental validity of XS5 over S5, and ‘s’ means incremental validity of S5 over either XS5.

### Discussion

The XS5 fared very similarly to the S5 on all three criteria on which they were compared. First, regarding correlations with longer personality measures, the XS5 performed markedly worse only on the Agreeableness dimension. For all other dimensions, the differences between the XS5 and S5 were negligible. The correlations were generally very high, with only the correlation for XS5 Agreeableness (*r* = .75) and Openness (r = .79) falling below *r* = .80 (that was also the average across the 5 domains). For comparison purposes, the average domain-level correlation between the 44-item BFI and the NEO PI-R has been reported as *r* = .69 [6]. Second, the self-peer correlations were only slightly higher for the S5 than the XS5, with all of the correlations exceeding .47. These correlations were also all very high; for comparison purposes, the average correlation between self-ratings and averaged ratings of two judges for the 300-item EPIP NEO has been reported as .49 [4]. Third, there were no differences between the S5 and the XS5 in predictive validity with regards to either self-reports of emotional experiences or behaviours.

The internal consistency reliabilities were generally modest, which is not unexpected given that we measured rather broad traits with a rather small number of items. The low reliability (alpha = .51) of the XS5 A scale raised some concerns. We noticed that this was due to the poor functioning of the Modesty facet, which was negatively correlated with some of the other Agreeableness facets. This item should be revised in future versions of the questionnaire. Even so, the intercorrelations between items were comparable to those in the longer scales; using Spearman-Brown prediction formula, the internal consistency of XS5 Agreeableness scale would be 0.81 using 24 items, and 0.89 using 48 items as in NEO PI-R.

The congruency coefficients generally indicated the principal solution of the XS5 was fairly similar to the intended target (congruence coefficients between .85 and.95 indicate a fairly similar factor structure [24]). The cross-country comparison of factor structures using ESEM indicated that in general, invariance of factor loadings cannot be assumed for neither XS5 nor NEO PI-R, and if one wishes to make conclusions that rest on invariance, one should test each relevant pair of languages separately. For one language pair on our study, English and German, the factorial invariance did hold. In addition to the similarity of languages and cultures (an idea which native speakers of those languages might object), the results may also reflect the degree of similarity of sampling procedures used in these countries. We failed to show factorial invariance in the Estonian—Finnish comparison, which may be partly due to differences in sampling procedures (e.g., Finnish sample was more heterogenous and, on the average, older). In addition, it was seen that the classical five-factor model is not sufficient to describe the data.

Controlling for acquiescence, either by partialling or subtraction, had very little effect on any of the results of Study 1. However, this cannot be considered very surprising; all our participants were university students, and controlling for acquiescence has been suggested to improve the psychometric properties of personality measures in representative samples, where acquiescence responding is more likely to be a problem [11–13]. We expected that controlling for acquiescence would have more of an impact in Study 2, in which we investigated the psychometric properties of the XS5 in a representative sample of Finns.

## Study 2

### Method

Participants completed the XS5 in conjunction with a survey that was conducted by a commercial survey company, Taloustutkimus Oy, on behalf of one of their clients. This client was primarily interested in the public perception of their products, and most items assessed respondents’ attitudes and preferences with regards to various consumer products. The survey company employed an internet panel that it had previously recruited and that was representative of the Finnish population in terms of age, sex, education, and internet use. Participants (N = 4916; 2492 females, mean age = 52.8; SD = 14.8, range 15–79, median = 55) were compensated for their time with lottery tickets (the prizes were mainly gift cards).

### Results

**Fig 3:**
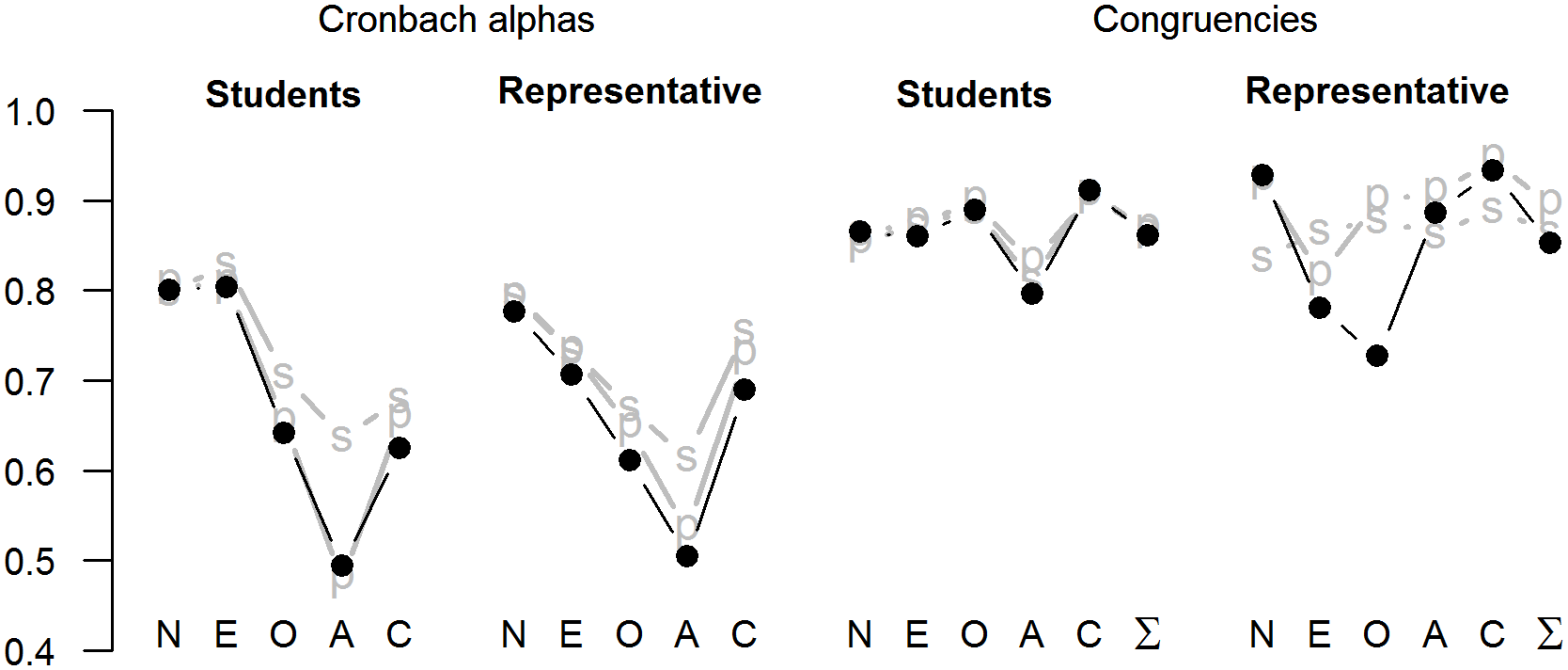
Cronbach alphas and congruence coefficients for two Finnish samples (student sample; representative internet sample). „p” = after partialling acquiescence; „s” = after subtracting acquiescence. Numeric data are presented in Tables G (student sample) and M (representative sample) in S1 File.

The internal consistency reliabilities of the XS5 were .78, .71, .61, .50, and .69, for ratings of N, E, O, A, and C respectively (Fig 3, Table M in S1 File). As in Study 1, the reliability of A was the lowest. Controlling for acquiescence (especially with subtraction method) improved the reliabilities substantially, having the strongest impact on the reliability of the A scale, which increased from .50 to .61.

The congruence coefficients for principal components of the XS5 were .93, .78, .73, .89, and .93, for N, E, O, A, and C, respectively (principal components loadings are reported in Table N in S1 File). Both the E scale and O scale failed to meet the criteria of fair similarity proposed by Lorenzo-Seva and colleagues [23]. As expected, controlling for acquiescence improved the situation considerably. Partialling for acquiescence resulted in congruence coefficients of .92, .82, .90, 91, and .95, and the subtraction method yielded .84, .87, .88, .86, and .89, respectively for N, E, O, A, and C. Controlling for acquiescence, the principal component structures became fairly similar with the intended target.

## General discussion

### Overview

In a previous paper [1], we have proposed an idea of “Comprehensive Single Items” (CSI) for constructing short questionnaires, where items are written to reflect expert definitions of each trait to be measured as closely as possible. The “Short Five” questionnaire was constructed to exemplify this idea, containing, for each of the subscales of the NEO PI-R, an item describing a person having a low score on that subscale, and another item describing a high scorer. This questionnaire performed quite well in terms of psychometric properties. Our purpose here was to examine the possibility of shortening this questionnaire up to the theoretical minimum—so that each subscale would contain only one item.

We did not set up to construct a perfect personality questionnaire: instead, our less aspiring goal was to construct a questionnaire that would be almost as good as the best ones available, but much shorter. The success of this goal is best assessed by the correlations between XS5 and more traditional, longer inventories: in this study, we mostly used NEO PI-R that is internationally recognized as the gold standard questionnaire measure of the Five Factor Model; EPIP-NEO as an analogue of NEO PI-R was used in the Estonian sample. Across samples, the correlations between the five-factor domain scales of XS5 and their longer counterparts ranged from 0.74 to 0.84. These figures are somewhat lower than the dependability coefficients of the NEO PI-R which in a recent meta-analysis [25] ranged from 0.88 to 0.92, suggesting that neither the S5 nor the XS5 can be considered exactly identical to the NEO PI-R. However, the gap is not large, and was even smaller in some countries: for example, in Finnish sample, the correlations between XS5 and NEO PI-R ranged from .84 to .90 which is only slightly less than the meta-analytic dependability coefficients of the NEO PI-R.

As a second comparison, in the Estonian sample, the correlations between XS5 and EPIP-NEO ranged from 0.83 to 0.90 when the full version of S5 was used and from 0.78 to 0.89 with the abbreviated XS5. In the study introducing the EPIP-NEO, its correlations with NEO PI-R were found to range from 0.83 to 0.90, thus not remarkably higher from those of XS5, and being in the same range as for full version of S5. Despite lower reliability, the XS5 is almost as “similar” to the NEO PI-R as the EPIP-NEO.

### Consensus and prediction of external criteria

Self-peer correlations have often been used as indicators of consensus on ratings of personality traits. Considering that consensus does not necessarily imply validity, and self-peer correlation is a particular type of consensus that is possibly influenced by several methodological issues (such as, in a typical study, self-selection bias), we have used consensus here only to compare XS5 to other inventories. High consensus does not imply truth in any absolute sense; however, if consensus is consistently and importantly higher using inventory A than inventory B, then we have a reason to prefer the first inventory over the second, all other things being equal. In our study, the self-peer correlations for XS5 domain scales ranged from 0.47 to 0.64, with the average of 0.53 (Table L in S1 File; cf. [1]). The corresponding figures for full-length S5 were: 0.50 to 0.67, averaging 0.56. In an earlier study [26] using NEO PI-R in a similar sample of students, the self-peer agreement correlations ranged from 0.43 to 0.67, averaging 0.56. We thus see almost no loss of consensus when S5 is shortened by half by methods described in the present paper. In addition, both S5 and XS5 exhibited self-peer consensus to approximately the same degree as NEO PI-R. Essentially, self-other consensus measures the degree to which an inventory captures the socially relevant and easily perceivable aspects of a trait in a reliable way. In this capacity, there was little difference between NEO PI-R, S5, and XS5, as long as we use domain scales (subscale level would need a more fine-grained analysis which is outside the scope of the present paper).

We investigated two types of external criteria: emotional experience, and self-reported behaviours. In terms of prediction of these criteria, using FFM domain scores, there were no big differences between S5 and XS5; we have previously shown [1] that longer scales (EPIP-NEO and NEO PI-R) did not have a clear advantage over the S5, and, if anything, that S5 tended to outperform the shortened versions of these longer scales. Thus again, in predicting external criteria using the FFM domain scales, there was little difference between NEO PI-R, S5, and XS5.

### Internal consistency

In terms of psychometric properties that are typically considered when evaluating the goodness of adaptations of personality inventories in the NEO PI-R tradition (i.e., internal reliabilities, and similarity of principal component structures), the performance of XS5 was acceptable but not excellent. Controlling for acquiescence, as recommended in some studies [13] did no harm, and was beneficial in the representative Finnish sample. However, goodness of the psychometric properties is not an end in itself: as controlling for acquiescence had no effect on the prediction of external criteria and had an inconsistent effect on self-peer consensus, we see little reason to recommend it as a general practice, at least with XS5. In addition, subtracting acquiescence as recommended by Rammstedt and colleagues [13] may produce an illusory enhancement in internal reliability by introducing an additional common component of variance in all items in all scales. Thus, even though our results are partly in accordance with the recommendations given by Rammstedt and colleagues, this issue needs some further research before the subtraction of acquiescence can be recommended as a part of a scoring system.

In interpreting the internal consistency statistics for XS5, one should take into account not only the number of items in the scales (which is considerably less than in traditional scales designed to measure similar constructs) but also the breadth of the traits to be measured. Constructing a highly internally consistent measure of a narrow trait such as self-esteem is a relatively straightforward task. The indicators of broader traits, such as neuroticism, need not be so strongly correlated with each other—that follows directly from the definition of “broad trait”. It is possible to some extent to measure the breadth of traits [27] but no such systematic attempts are known to the present authors. Thus, the maximum possible level of internal reliability of a scale depends on a partly unknown factor.

In addition, as Nunnally and Bernstein [28] have argued (p. 265), the choice of required level of reliability depends on our purpose in using the test: “In the early stages of research… one saves time and energy by working with instruments that have only modest reliability, for which purpose reliabilities of .70 or higher will suffice. In contrast to the standards in basic research, in many applied settings a reliability of .80 is not nearly high enough.” This is a good description of the intended uses of XS5, although we would argue for replacing, in the above quote, “.70 or higher” by “.70 or even lower, depending on the breadth of the construct and available time”.

### Controlling for acquiescence

The principle of comprehensive single items (CSI) seemingly suggests a single item per scale; however, in constructing the S5, it was decided to use two items in each subscale—partly because of an ambiguity as to which end of each dimension should be chosen, and partly as an attempt to cancel out acquiescent responding. In XS5, we have used a complex set of criteria to choose one item from among the two in each subscale. This has an obvious effect on balanced keying: whereas in S5, each subscale is pairwise balanced (i.e., each item has an exact counterpart with opposite meaning), in XS5, there is balancing in blocks but not in pairs (i.e., in each domain scale, there is an equal number of positively and negatively keyed items but none of the item has a counterpart with exactly opposite meaning). An equivalent form of balancing is used in most of the longer personality inventories such as NEO PI-R, both at the level of subscales (“facets”) as well as the FFM domain scales. In S5, thus, acquiescence can be assessed much more precisely, and at the item level, whereas in XS5, one can only obtain a general score. Conversely, controlling for acquiescence is more important in XS5, as it is not cancelled out by balanced keying. A good process model of acquiescence [29] might help solve this dilemma. From a psychometric point of view, there is not much evidence about whether acquiescence is better conceptualized to operate at the level of single items with specific meaning and should thus be measured using item pairs with directly opposite meaning, or as a general tendency, which can just as well be measured using item blocks with roughly opposite meaning. From the present results, one can see that acquiescence as measured in in the latter way does not help much in deconfounding useful and useless variance in personality tests.

### Measurement models and short scales: Questions for further research

There are a couple of interesting questions that we would have liked to investigate here, but that we deliberately chose to set aside for future: conformity of the XS5 structure to theory-based explanatory models of personality trait covariation, possible cross-cultural differences in structure, and measurement equivalence across cultures. We believe these are important questions that merit further investigation. An intent to keep the paper focused (shortening the S5 questionnaire while keeping its usefulness) was our main reason for not delving into these questions, but it was not the only one.

These questions would be typically answered by means of a version of confirmatory factor analysis (CFA). In our view, the differences between confirmatory and exploratory methods have been exaggerated in the literature,—the way CFA is typically used in literature, may include exploratory steps (for example, “perfecting” the model based on modification indexes) as well as subjective judgments of satisfactoriness of the degree of fit based on rules of thumb and comparative fit indexes. Secondly, we are not convinced that the reflective latent variable models are true for personality questionnaires, even if they are often useful approximations. These are solvable questions, but have no quick and easy answer.

Our main purpose in the present paper was to show that by using comprehensive single items (CSI) one can construct questionnaires that are promising alternatives to longer inventories, even when a single item is used per subscale. This is evidenced by fair correlations with longer scales, as well as comparable external validity. We expected and found some degree, but not perfect, structural similarity with longer scales; we did not expect a good fit with a theoretical model. The fit of personality inventories to simple theoretical latent-trait models is rarely satisfactory; see next section for our account of the main reasons why this is the case. Although exploratory structural equation modelling (ESEM) has given more promising results [30], it is less theory-based and thus offers less in addition to the traditional descriptive methods such as PCA that was used here for comparability with the large literature on NEO PI-R.

We believe that cross-cultural equivalence comparisons have their value, especially as the results, if interpreted properly, can help making better adaptations or constructing more easily adaptable questionnaires. On the other hand, we see no reason why correlational structure of personality traits should be exactly equal in all countries. When the items of the different language versions of a questionnaire are made, content-wise, as “equal” as possible (comparing all pairs of languages for all conceivable nuances of meaning), then structural dissimilarities might inform us about genuine cultural differences (cf. [31]). We have made some steps in this direction with S5, but by no means exhausted all possibilities—therefore, the result of any cross-language structural comparison will be ambiguous, especially for some language pairs such as Estonian and Chinese where none of the present authors can make direct comparisons.

Reflective latent trait models have, during recent decades, become increasingly popular in research on personality. We would like to point out that these models have shortcomings besides their undeniable merits. For example, the idea of interchangeability of indicators, going back to classical test theory [32], is simply not true. Consider an extraversion questionnaire consisting of items of talkativeness and friendliness: the scale’s meaning will change considerably if we “interchange” away all friendliness items and replace them with talkativeness items. Usually, the idea is to follow a “map” of the content area so that all “regions” of extraversion would be covered. However, the idea of content mapping, based on the notion of content validity [33] is not consistent with interchangeability of indicators.

As a second example, reflective models presume that indicators are caused by latent constructs. Multivariate statistics is not a miraculous way of testing causality without having an experimental (or at least, quasi experimental, in whatever sense) design. Thus, before using a reflective model, we should be convinced that it at least makes sense to think of each and every latent variable as a causal unit. Even admitting such a hypothesis, one will immediately see that every conceivable response to a personality item has multiple causes. For example, two items, one in Openness to actions and another in Impulsiveness subscale may share a common theme: both are related to eating. This makes them covary, and could be represented as residual correlation. Including many such covariations in a model, however, is against best practices, and will make an ugly, atheoretical model which is not, in essence, different from a descriptive picture. On the other hand, information from residual correlations could (and should) be used to get ideas of how to write better items or improve cross-cultural comparability of items.

In summing up, while we see many potential uses of SEM and related techniques in constructing better questionnaires, we do not agree that pure and simple reflective models are realistic as measurement models for personality questionnaires. While network models might represent a more realistic alternative [34], they are, at least in their current form, not of immediate help in analysing cross-sectional questionnaire data.

Regardless of the above considerations, we ran a test of factorial invariance and concluded that caution is needed in interpreting cross-cultural comparisons using XS5. In our study, we failed to confirm factorial invariance for all 6 countries involved, but could confirm it for Germany and UK. This non-invariance was not specific to XS5: the same pattern of results held for NEO PI-R as well. On the other hand, one should also exercise caution when interpreting the results on cross-cultural invariance. Our study was neither designed nor optimized for cross-cultural comparisons: different sampling procedures were used in each country.

### Concluding remarks

In several places above, we have concluded that XS5 is more or less equivalent to S5, as well as longer personality scales. We stand by this conclusion as far as the FFM domain scales are concerned; when subscales are essential, we would still recommend using the full version of S5. First of all, any differences in reliability are much larger when we compare a single item to two items, than when comparing a 6-item scale to a 12-item scale. Therefore, reliability differences between S5 and XS5 are much more marked at the level of subscales than at the level of domain scales. Secondly, the effect of acquiescence is more or less cancelled out in XS5 domain scales, but not in the subscales. At the same time, the XS5 subscales do contain useful information and can be used to complement the domain scales when needed.

Finally, can make it even shorter, say, 10 items instead of 30? This is a question of purpose, not of principle. Our main intention with S5 was one-to-one correspondence with each subscale of NEO PI-R; this also allows a good correspondence at the domain level. Using the CSI approach, one can ask about rather general traits but there is a limit to the level of abstractness that we can expect from the respondents. The FFM domains are too general and abstract to include in a comprehensive single item; this would either be a tediously long and “multi-barreled” item that is difficult to respond, or else it would leave out too many important aspects and the result would not be comparable with the long inventory. On the other hand, the NEO PI-R subscales (such as activity, or positive emotionality) seem to represent more or less the “basic” level of hierarchy [27] that is easily understandable to the respondents. Thus, reducing the number of items to less than 30 would necessitate a different system of subscales, and would thus be likely to reduce the comparability with NEO PI-R.

## Supporting information

S1 Appendix Instructions and items of the XS5. (PDF)

S1 File Supplementary results. (PDF)

S1 Data Data files and description of the variables. (ZIP)

## References

[1] Konstabel K, Lönnqvist J-E, Walkowitz G, Konstabel K, Verkasalo M (2012) The "Short Five" (S5): Measuring personality traits using comprehensive single items. Eur J Pers 26: 13–29.

[2] John OP, Srivastava S 1999 The Big Five Trait taxonomy: History, measurement, and theoretical perspectives In Pervin LA & John OP, editors. Handbook of personality: Theory and research (2nd ed., pp. 102–139). New York: Guilford.

[3] Costa PT Jr, McCrae RR 1992 Revised NEO Personality Inventory (NEO PI-R) and NEO Five-Factor Inventory (NEO-FFI) professional manual. Odessa, FL: Psychological Assessment Resources, Inc.

[4] Mõttus R, Pullmann H, Allik J 2006 Towards more readable Big Five personality inventories. Eur J Psychol Assess 22: 149–157.

[5] Rammstedt B, John OP 2007 Measuring personality in one minute or less: A 10 item short version of the Big Five Inventory in English and German. J Res Pers 41: 203–212.

[6] Gosling SD, Rentfrow PJ, Swann WB Jr. 2003 A Very Brief Measure of the Big Five Personality Domains. J Res Pers 37: 504–528.

[7] Konstabel K, Virkus A 2006 How similar are the conceptual and empirical structures of personality traits? Eur J Pers 20: 337–353.

[8] McCrae RR, Herbst JH, Costa PT Jr 2001 Effects of acquiescence on personality factor structures In Riemann R, Ostendorf F, & Spinath F, editors. Personality and temperament: Genetics, evolution, and structure (pp. 217–231). Berlin: Pabst Science Publishers.

[9] Herzberg PY, Brähler E 2006 Assessing the Big-Five personality domains via short forms: A cautionary note and a proposal. Eur J Psychol Assess 22: 139–148. http://dx.doi.org/10.1027/1015-5759.22.3.139

[10] McCrae RR, Costa PT Jr 2007 Brief versions of the NEO-PI-3. J Individ Differ, 28: 116–128.

[11] Rammstedt B, Goldberg LR, Borg I 2010 The measurement equivalence of Big Five factor markers for persons with different levels of education. J Res Pers 44: 53–61. doi: 10.1016/j.jrp.2009.10.005 20401177 10.1016/j.jrp.2009.10.005 PMC2854544

[12] Rammstedt B, Kemper 2011 Measurement equivalence of the Big Five: Shedding further light on potential causes of the educational bias. J Res Pers 45: 121–125.

[13] Rammstedt B, Kemper CJ, Borg I 2013 Correcting Big Five personality measurements for acquiescence: An 18-country cross-cultural study. Eur J Pers 27: 71–81.

[14] Ten Berge JMF 1999 Factor similarity measures In Read CB, Banks DL & Kotz S, editors. Encyclopedia of Statistical Sciences (Update Vol. 3; pp. 239–240). New York: John Wiley & Sons.

[15] Lönnqvist J-E, Paunonen S, Tuulio-Henriksson A, Lönnqvist J, Verkasalo M 2007 Substance and Style in Socially Desirable Responding. J Pers 75: 291–322. doi: 10.1111/j.1467-6494.2006.00440.x 17359240 10.1111/j.1467-6494.2006.00440.x

[16] Ostendorf F, Angleitner A 2004 NEO-Persönlichkeitsinventar nach Costa und McCrae, Revidierte Fassung [NEO Personality Inventory after Costa and McCrae, Revised]. Göttingen, Germany: Hogrefe.

[17] Pando AC, Pamos A, Cubero NS, Costa PT Jr. 1999 Inventario de personalidad neo revisado (neo pi-r), inventario neo reducido de cinco factores (neo-ffi): manual profesional. Madrid, Spain: Tea Ediciones.

[18] McCrae RR, Costa PT Jr, Yik MSM 1996 Universal aspects of Chinese personality structure In Bond MH, editor. The handbook of Chinese psychology (pp. 189–207). Hong Kong: Oxford University Press.

[19] Goldberg LR 1999 A broad-bandwidth, public-domain, personality inventory measuring the lower-level facets of several five-factor models In Mervielde I, Deary I, De Fruyt F & Ostendorf F, editors. Personality Psychology in Europe (Vol. 7 pp. 7–28). Tilburg: Tilburg University Press.

[20] Goldberg LR, Johnson JA, Eber HW, Hogan R, Ashton MC, Cloninger CR, Gough HC 2006 The International Personality Item Pool and the future of public-domain personality measures. J Res Pers 40: 84–96.

[21] Paunonen V 1998 Hierarchical organization of personality and prediction of behavior. J Pers Soc Psychol, 74, 538–556.

[22] Bryant FB, Satorra A 2012 Principles and practice of scaled difference chi-square testing. Structural Equation Modeling, 19: 372–398.

[23] Watson D 2000 Mood and Temperament. New York: Guilford.

[24] Lorenzo-Seva U, ten Berge JMF 2006 Tucker's congruence coefficient as a meaningful index of factor similarity. Methodology (Gott) 2: 57–64.

[25] Gnambs T 2014 A meta-analysis of dependability coefficients (test—retest reliabilities) for measures of the Big Five. J Res Pers 52: 20–28.

[26] Konstabel K, Aavik T, Allik J 2006 Social desirability and consensual validity of personality traits. Eur J Pers 20: 549–566.

[27] John OP, Hampson SE, Goldberg LR 1991 The basic level in person ality-trait hierarchies: Studies of trait use and accessibility in different contexts. J Pers Soc Psych 60: 348–361 10.1037//0022-3514.60.3.348 2027078

[28] Nunnally JC, Bernstein IH 1994 Psychometric Theory (3^rd^ ed). New York: McGraw Hill.

[29] Knowles ES, Condon CA 1999 Why people say" yes": A dual-process theory of acquiescence. J Pers Soc Psychol 77: 379–386.

[30] Marsh HW, Lüdtke O, Muthén B, Asparouhov T, Morin AJ, Trautwein U, Nagengast B 2010 A new look at the big five factor structure through exploratory structural equation modelling. Psychol Assess 22: 471–91. 20822261 10.1037/a0019227

[31] Konstabel K, Realo A, Kallasmaa T 2002 Exploring the sources of variations in the structure of personality traits across cultures In McCrae RR & Allik J (Eds) The Five-Factor Model of Personality Across Cultures (pp. 29–52). New York: Kluwer Academic Publishers

[32] Borsboom D 2005 Measuring the mind: Conceptual issues in contemporary psychometrics. Cambridge: Cambridge University Press.

[33] Haynes SN, Richard DCS, Kubany ES 1995 Content validity in psychological assessment: A functional approach to concepts and methods. Psych Assessement 7: 238–247.

[34] Schmittmann VD, Cramer AOJ, Waldorp LJ, Epskamp S, Kievit RA, Borsboom D 2011 Deconstructing the construct: a network perspective on psychological phenomena. New Ideas in Pscyhology, 31:43–53.

