# Supplementary material for: Measuring single constructs by single items: Constructing an even shorter version of the “Short Five” personality inventory
Source: PLoS One. 2017 Aug 11;12(8):e0182714. doi: 10.1371/journal.pone.0182714 (PMC5553894; doi:10.1371/journal.pone.0182714)
Supplement: S1 Appendix — (PDF) [file pone.0182714.s001.pdf]

S1 Appendix
Instructions and items of XS5

Instructions

Please read the following statements and indicate to what extent they apply to you. Write the suitable number in the box in front of the descriptive statement. Use the following scale:

The description is…

-3	-2	-1	0	1	2	3
**Completely wrong**	**Mostly wrong**	**More wrong than right**	**Neutral; neither right nor wrong**	**More right than wrong**	**Mostly right**	**Completely right**

In some cases you may want to give different answers to different parts of an item. In these case try to find an "average answer" giving more weight to the part(s) that you consider more important. For example, if you think the start of an item is "mostly right" about you but the end is "more wrong than right" then pick one of the options in between them (0 or 1).

Items

Items are listed in the order they were presented in the questionnaire. The abbreviations of the subscales are given in parentheses after each item, followed by an asterisk for reverse keyed items.

1. I am often nervous, fearful, and anxious, and worry that something might go wrong. (N1)

2. I like people; I am friendly and open talking to strangers. (E1)

3. I have a vivid imagination. I like to fantasize and let my thoughts run free. (O1)

4. I trust people and I believe that everyone is honest and has good intentions most of the time. (A1)

5. I am a serious rather than a cheerful person. I have rarely been overflowing with joy. (E6*)

6. I have quite traditional values; I am considered to be somewhat close-minded when it comes to the values of other cultures and other groups of people. (O6*)

7. I often rush into action without considering the consequences of my actions and decisions. (C6*)

8. I enjoy meeting and associating with a lot of people. I take pleasure in the company of others - the more people, the better. (E2)

9. I have a deep appreciation for fine arts and beauty. I am much impressed by and interested in music, poetry, and art. (O2)

10. I am a methodical person and I love cleanliness and order. I want every thing to be in its right place. (C2)

11. I am not looking for excitement or adventures. I do not like to take risks. (E5*)

12. I am not interested in abstract or theoretical ideas. In my mind, ideas without practical applications are a waste of time. (O5*)

13. I often postpone difficult or unpleasant activities and leave things unfinished. It is difficult for me to pull myself together and do the things that I have to. (C5*)

14. I am a reliable person, who values ethical principles; I keep my promises and work carefully and thoroughly. (C3)

15. I feel comfortable around other people; Most of the time I am not bothered by teasing or by embarrassing situations. (N4*)

16. I am a stubborn person who often gets into arguments; I openly express my anger or my dislike for someone. (A4*)

17. I am active and I like to keep myself busy; I often feel bursting with energy. (E4)

18. I like to try different activities, to visit different places, to try out unfamiliar and exotic things from time to time; I love novelty and variety. (O4)

19. I know for certain what I want to accomplish and I work hard for it. (C4)

20. I rarely feel hopeless; I do not tend to blame myself needlessly. In general, I am content with myself and my life. (N3*)

21. I prefer to remain unnoticed in the background. I often let other people talk and decide things on my behalf. (E3*)

22. My feelings are not important to me; most of the time I do not pay any attention to them. (O3*)

23. I do not want to deal with other people's problems; I am considered a selfish and egotistical person. (A3*)

24. It is very difficult for me to resist temptation and to keep my desires and feelings in check; I do things that I regret later. (N5)

25. I do not want to be in the centre of attention. I do not like to talk about myself or my accomplishments. (A5)

26. I am a well composed person and it is difficult to upset or anger me. (N2*)

27. I believe that honesty does not take one very far in life. When necessary, I try to take advantage of others. (A2*)

28. I often feel helpless and indecisive, especially in complicated situations. I easily lose my nerve when I feel that I cannot cope. (N6)

29. I believe that every person deserves respect. I feel compassion for those people who have been less lucky in life than I have. (A6)

30. I often feel that I am not competent enough to do something; I am not very productive and effective in my work. (C1*)
