# Supplementary material for: Measuring single constructs by single items: Constructing an even shorter version of the “Short Five” personality inventory
Source: PLoS One. 2017 Aug 11;12(8):e0182714. doi: 10.1371/journal.pone.0182714 (PMC5553894; doi:10.1371/journal.pone.0182714)
Supplement: S1 File — (PDF) [file pone.0182714.s002.pdf]

S2. Supplementary information to

Measuring single constructs by single items: Constructing an even shorter version of the "Short Five" personality inventory

Kenn Konstabel, Jan-Erik Lönnqvist, Sointu Leikas, Regina García Velázquez, Hiaying Qin, Markku Verkasalo, Gari Walkowitz

Table A. Response options for the multi-barreledness questionnaire 2

Table B. Preference z-scores for S5 items. Positive scores indicate a preference of positively keyed item over the negative one, according to the respective criterion 3

Table C. All possible choices of three positive and three negative subscales across the five-factor model domains, and the corresponding preference z-scores 4

Table D. Internal consistency reliabilities (Cronbach alphas) by countries, with and without controlling for acqueiscence 5

Table E. Congruence coefficients (after Procrustes rotation targeted towards US normative structure of the NEO PI-R) by country 6

Table F. Correlations with EPIP-NEO: Estonia 7

Table G. Correlations with NEO PI-R: Finland (student sample) 8

Table H. Correlations with NEO PI-R: UK 9

Table I. Correlations with NEO PI-R: Germany 10

Table J. Correlations with NEO PI-R: Spain 11

Table K. Correlations with NEO PI-R: China 12

Table L. Self-peer correlations: Estonia 13

Table M. Internal consistencies (Cronbach alphas) in Finnish representative sample 14

Table N. Principal component structure in the Finnish representative sample (rotated towards the US normative NEO PI-R structure using Procrustes rotation) 15

Table O. Principal component structure of XS5 in the Spanish sample (rotated towards the US normative NEO PI-R structure using Procrustes rotation) 16

Table P. Principal component structure of the 60-item S5 in the Spanish sample (rotated towards the US normative NEO PI-R structure using Procrustes rotation) 17

Table Q. Principal component structure of the XS5 in the Chinese sample (rotated towards the US normative NEO PI-R structure using Procrustes rotation) 18

Table R. Principal component structure of the 60-item S5 in the Chinese sample (rotated towards the US normative NEO PI-R structure using Procrustes rotation) 19

Table S. Fit statistics for the ESEM models (XS5) in each country 20

Table T. Fit statistics for the ESEM models (NEO PI-R) in each country 21

Table A. Response options for the multi-barreledness questionnaire

Response option	Label
0	No contradiction, the item is easy to respond to
1	If one reads the item several times, some part of the item can be interpreted as contradictory with some other part.
2	Some part of the item is in slight contradiction with another part.
3	The item asks about more than one thing, this may make it difficult for some people to respond to the item.
4	The item asks about more than one thing, this would make it difficult for me to respond to the item.
5	No-one can respond to this item unambiguously, too many different things are asked about.

Note. The exact instructions for ratings the multi-barreledness of responses were: Here are 60 items from the S5 personality inventory. Some of the items may ask about two or more things and may therefore be difficult to respond to. Read each item and indicate to what extent it would be difficult for you to respond to the item.

Table B. Preference z-scores for S5 items. Positive scores indicate a preference of positively keyed item over the negative one, according to the respective criterion

	CR1	CR2	CR3	Mean	Sel
N1	0.47	-0.38	1.34	0.48	*
N2	-2.11	-0.60	0.09	-0.87	
N3	0.55	-0.25	-1.57	-0.42	
N4	0.66	-0.82	1.10	0.31	
N5	0.25	1.11	-0.09	0.42	*
N6	0.21	0.23	0.79	0.41	*
E1	-0.11	0.41	-0.32	-0.01	*
E2	1.13	2.01	0.01	1.05	*
E3	1.16	0.15	-2.70	-0.46	
E4	0.43	-0.32	0.19	0.10	*
E5	-0.90	-0.49	0.05	-0.45	
E6	-0.45	-0.89	0.42	-0.31	
O1	0.56	0.09	0.32	0.32	*
O2	0.22	1.67	-1.35	0.18	*
O3	0.20	1.35	-1.68	-0.05	
O4	0.17	1.16	-0.30	0.34	*
O5	1.29	0.22	-1.00	0.17	
O6	-1.30	-1.25	1.17	-0.46	
A1	0.37	-0.58	0.18	-0.01	*
A2	-1.73	-1.60	0.95	-0.79	
A3	-0.49	-0.97	-0.74	-0.73	
A4	-0.32	0.06	-0.70	-0.32	
A5	-1.78	1.41	0.94	0.19	*
A6	2.09	1.51	1.05	1.55	*
C1	-0.92	-1.06	0.14	-0.61	
C2	1.02	0.48	1.28	0.93	*
C3	-0.07	-0.43	1.15	0.22	*
C4	1.03	-0.66	-0.13	0.08	*
C5	-1.36	0.29	-0.99	-0.68	
C6	-0.30	-1.84	0.40	-0.58	

Note. CR1 = correlations with longer questionnaires (NEO PI-R or EPIP-NEO), z-transformed within country, weighted by sample size, and averaged. CR2 = self-peer and peer-peer agreement combined. CR3 = multi-barreledness ratings. Mean = average of CR1, CR2, and CR3. Sel = asterisks indicate the subscales where positively keyed item is chosen in the optimal subset.

Table C. All possible choices of three positive and three negative subscales across the five-factor model domains, and the corresponding preference z-scores

Combination	N	E	O	A	C
123	-1.96	1.23	0.40	-2.95	1.71
124	-0.50	**2.36**	**1.18**	-2.13	1.43
125	-0.27	1.26	0.84	-1.11	-0.09
126	-0.30	1.54	-0.42	1.61	0.12
134	0.41	-0.67	0.73	-2.01	0.02
135	0.63	-1.76	0.38	-0.99	-1.50
136	0.61	-1.48	-0.88	1.73	-1.29
145	2.10	-0.63	1.16	-0.16	-1.78
146	2.07	-0.36	-0.10	2.56	-1.57
156	**2.30**	-1.45	-0.45	**3.58**	-3.09
234	-2.30	1.45	0.45	-3.58	**3.09**
235	-2.07	0.36	0.10	-2.56	1.57
236	-2.10	0.63	-1.16	0.16	1.78
245	-0.61	1.48	0.88	-1.73	1.29
246	-0.63	1.76	-0.38	0.99	1.50
256	-0.41	0.67	-0.73	2.01	-0.02
345	0.30	-1.54	0.42	-1.61	-0.12
346	0.27	-1.26	-0.84	1.11	0.09
356	0.50	-2.36	-1.18	2.13	-1.43
456	1.96	-1.23	-0.40	2.95	-1.71
Max	2.30	2.36	1.18	3.58	3.09

Note. The numbers in the first column (“Combination”) denote subscales where a positively keyed item is selected for the abridged version. As an example, in the first row (“123”), positively keyed items are chosen to represent the first three subscales of each domain (N1, N2, and N3; E1, E2, and E3, etc), and consequently, negatively keyed items are chosen to represent the rest of the subscales (N4, N5, and N6, etc.)

Table D. Internal consistency reliabilities (Cronbach alphas) by countries, with and without controlling for acqueiscence

	N	E	O	A	C
Estonia	0.76	0.81	0.54	0.55	0.68
Finland	0.80	0.80	0.64	0.49	0.62
UK	0.76	0.57	0.68	0.57	0.69
Germany	0.74	0.75	0.65	0.58	0.70
Spain	0.68	0.72	0.57	0.38	0.61
China	0.75	0.77	0.61	0.50	0.62
Partialling acquiescence					
Estonia	0.76	0.82	0.57	0.57	0.69
Finland	0.81	0.81	0.65	0.48	0.66
UK	0.78	0.63	0.70	0.61	0.72
Germany	0.75	0.77	0.67	0.60	0.72
Spain	0.65	0.74	0.59	0.42	0.64
China	0.76	0.79	0.63	0.52	0.64
Subtracting acquiescence					
Estonia	0.75	0.81	0.60	0.64	0.74
Finland	0.80	0.83	0.71	0.64	0.68
UK	0.75	0.63	0.74	0.62	0.74
Germany	0.73	0.76	0.65	0.60	0.73
Spain	0.70	0.73	0.59	0.42	0.64
China	0.77	0.79	0.64	0.52	0.65

Table E. Congruence coefficients (after Procrustes rotation targeted towards US normative structure of the NEO PI-R) by country

	N	E	O	A	C	Matrix
Estonia	0.93	0.91	0.84	0.91	0.93	0.91
Finland	0.87	0.86	0.89	0.80	0.91	0.86
UK	0.92	0.91	0.89	0.92	0.94	0.91
Germany	0.89	0.82	0.73	0.88	0.86	0.83
Spain	0.88	0.86	0.84	0.84	0.91	0.87
China	0.89	0.84	0.83	0.75	0.81	0.82
Partialling acquiescence						
Estonia	0.92	0.91	0.82	0.93	0.93	0.90
Finland	0.85	0.87	0.90	0.83	0.91	0.87
UK	0.90	0.88	0.92	0.91	0.93	0.91
Germany	0.88	0.80	0.75	0.86	0.84	0.83
Spain	0.86	0.86	0.84	0.85	0.91	0.86
China	0.88	0.85	0.86	0.76	0.85	0.84
Subtracting acquiescence						
Estonia	0.90	0.90	0.84	0.88	0.94	0.90
Finland	0.87	0.87	0.89	0.81	0.91	0.87
UK	0.89	0.88	0.82	0.91	0.93	0.89
Germany	0.85	0.80	0.70	0.83	0.89	0.81
Spain	0.87	0.86	0.85	0.86	0.91	0.87
China	0.88	0.85	0.88	0.76	0.85	0.84

Table F. Correlations with EPIP-NEO: Estonia

	s5	xs5	xs5part	xs5subtr
Anxiety (N1)	0.72	0.63	0.63	0.63
Angry Hostility (N2)	0.79	0.76	0.76	0.73
Depression (N3)	0.77	0.68	0.68	0.65
Self-Consciousness (N4)	0.66	0.59	0.60	0.55
Impulsiveness (N5)	0.66	0.63	0.53	0.60
Vulnerability (N6)	0.71	0.62	0.60	0.61
Warmth (E1)	0.79	0.74	0.73	0.73
Gregariousness (E2)	0.79	0.73	0.72	0.73
Assertiveness (E3)	0.74	0.60	0.64	0.55
Activity (E4)	0.67	0.57	0.55	0.56
Excitement Seeking (E5)	0.77	0.73	0.73	0.69
Positive Emotions (E6)	0.85	0.78	0.78	0.75
Openness to Fantasy (O1)	0.82	0.78	0.78	0.77
Openness to Aesthetics (O2)	0.82	0.78	0.79	0.79
Openness to Feelings (O3)	0.79	0.72	0.72	0.70
Openness to Actions (O4)	0.76	0.67	0.66	0.66
Openness to Ideas (O5)	0.61	0.47	0.44	0.47
Openness to Values (O6)	0.42	0.39	0.39	0.38
Trust (A1)	0.78	0.74	0.74	0.75
Straightforwardness (A2)	0.72	0.67	0.64	0.66
Altruism (A3)	0.76	0.65	0.65	0.63
Compliance (A4)	0.60	0.50	0.48	0.50
Modesty (A5)	0.76	0.62	0.62	0.63
Tender-Mindedness (A6)	0.53	0.59	0.61	0.61
Competence (C1)	0.66	0.57	0.55	0.57
Order (C2)	0.83	0.81	0.82	0.82
Dutifulness (C3)	0.75	0.59	0.62	0.65
Achievement Striving (C4)	0.75	0.71	0.71	0.71
Self-Discipline (C5)	0.77	0.70	0.68	0.68
Deliberation (C6)	0.83	0.77	0.70	0.76
Neuroticism	0.86	0.84	0.81	0.79
Extraversion	0.90	0.89	0.87	0.84
Openness	0.86	0.81	0.81	0.77
Agreeableness	0.83	0.78	0.76	0.75
Conscientiousness	0.89	0.86	0.84	0.83

Note. S5 = full (60-item) version of the “Short Five”. XS5 = abbreviated (30-item) version of the “Short Five”. Correlations between XS5 and EPIP-NEO after partialling for (xs5part) or subtracting acquiescence are shown in the two rightmost columns.

Table G. Correlations with NEO PI-R: Finland (student sample)

	s5	xs5	xs5part	xs5subtr
Anxiety (N1)	0.81	0.81	0.81	0.81
Angry Hostility (N2)	0.74	0.72	0.74	0.68
Depression (N3)	0.84	0.71	0.70	0.68
Self-Consciousness (N4)	0.81	0.64	0.64	0.62
Impulsiveness (N5)	0.81	0.76	0.71	0.75
Vulnerability (N6)	0.71	0.69	0.69	0.69
Warmth (E1)	0.63	0.51	0.51	0.50
Gregariousness (E2)	0.85	0.72	0.73	0.74
Assertiveness (E3)	0.84	0.74	0.76	0.71
Activity (E4)	0.78	0.61	0.61	0.61
Excitement Seeking (E5)	0.65	0.53	0.53	0.53
Positive Emotions (E6)	0.75	0.65	0.66	0.62
Openness to Fantasy (O1)	0.80	0.79	0.79	0.79
Openness to Aesthetics (O2)	0.77	0.78	0.78	0.78
Openness to Feelings (O3)	0.71	0.64	0.64	0.61
Openness to Actions (O4)	0.64	0.60	0.61	0.61
Openness to Ideas (O5)	0.69	0.55	0.58	0.52
Openness to Values (O6)	0.65	0.57	0.51	0.57
Trust (A1)	0.85	0.78	0.77	0.79
Straightforwardness (A2)	0.72	0.70	0.66	0.69
Altruism (A3)	0.69	0.61	0.59	0.59
Compliance (A4)	0.72	0.67	0.63	0.67
Modesty (A5)	0.69	0.52	0.51	0.51
Tender-Mindedness (A6)	0.70	0.64	0.64	0.64
Competence (C1)	0.63	0.53	0.50	0.53
Order (C2)	0.77	0.71	0.69	0.71
Dutifulness (C3)	0.61	0.58	0.59	0.58
Achievement Striving (C4)	0.72	0.62	0.60	0.63
Self-Discipline (C5)	0.81	0.78	0.76	0.77
Deliberation (C6)	0.82	0.77	0.77	0.74
Neuroticism	0.90	0.89	0.87	0.86
Extraversion	0.90	0.86	0.85	0.84
Openness	0.85	0.84	0.84	0.80
Agreeableness	0.84	0.77	0.74	0.73
Conscientiousness	0.86	0.83	0.82	0.80

Note. S5 = full (60-item) version of the "Short Five". XS5 = abbreviated (30-item) version of the "Short Five". Correlations between XS5 and NEO PI-R after partialling for (xs5part) or subtracting acquiescence are shown in the two rightmost columns.

Table H. Correlations with NEO PI-R: UK

	S5	XS5	xs5part	subtr
Anxiety (N1)	0.76	0.71	0.69	0.71
Angry Hostility (N2)	0.75	0.69	0.70	0.67
Depression (N3)	0.80	0.72	0.72	0.68
Self-Consciousness (N4)	0.65	0.50	0.53	0.46
Impulsiveness (N5)	0.66	0.59	0.55	0.58
Vulnerability (N6)	0.82	0.72	0.70	0.72
Warmth (E1)	0.78	0.72	0.68	0.70
Gregariousness (E2)	0.73	0.65	0.65	0.66
Assertiveness (E3)	0.69	0.56	0.56	0.55
Activity (E4)	0.66	0.64	0.65	0.65
Excitement Seeking (E5)	0.47	0.43	0.44	0.41
Positive Emotions (E6)	0.82	0.71	0.72	0.69
Openness to Fantasy (O1)	0.78	0.70	0.67	0.69
Openness to Aesthetics (O2)	0.86	0.81	0.79	0.80
Openness to Feelings (O3)	0.77	0.71	0.71	0.69
Openness to Actions (O4)	0.69	0.67	0.67	0.67
Openness to Ideas (O5)	0.76	0.60	0.60	0.58
Openness to Values (O6)	0.50	0.47	0.47	0.46
Trust (A1)	0.82	0.77	0.77	0.78
Straightforwardness (A2)	0.57	0.56	0.57	0.54
Altruism (A3)	0.66	0.59	0.61	0.55
Compliance (A4)	0.71	0.61	0.63	0.59
Modesty (A5)	0.72	0.58	0.54	0.56
Tender-Mindedness (A6)	0.64	0.60	0.59	0.59
Competence (C1)	0.58	0.48	0.46	0.48
Order (C2)	0.77	0.73	0.74	0.74
Dutifulness (C3)	0.59	0.57	0.58	0.58
Achievement Striving (C4)	0.79	0.76	0.77	0.77
Self-Discipline (C5)	0.80	0.76	0.76	0.74
Deliberation (C6)	0.73	0.61	0.63	0.58
Neuroticism	0.89	0.86	0.84	0.82
Extraversion	0.87	0.84	0.84	0.81
Openness	0.87	0.83	0.83	0.80
Agreeableness	0.83	0.80	0.80	0.74
Conscientiousness	0.89	0.86	0.86	0.82

Note. S5 = full (60-item) version of the “Short Five”. XS5 = abbreviated (30-item) version of the “Short Five”. Correlations between XS5 and NEO PI-R after partialling for (xs5part) or subtracting acquiescence are shown in the two rightmost columns.

Table I. Correlations with NEO PI-R: Germany

	S5	XS5	Xs5Part	Subtr
Anxiety (N1)	0.76	0.71	0.69	0.71
Angry Hostility (N2)	0.75	0.69	0.70	0.67
Depression (N3)	0.80	0.72	0.72	0.68
Self-Consciousness (N4)	0.65	0.50	0.53	0.46
Impulsiveness (N5)	0.66	0.59	0.55	0.58
Vulnerability (N6)	0.82	0.72	0.70	0.72
Warmth (E1)	0.78	0.72	0.68	0.70
Gregariousness (E2)	0.73	0.65	0.65	0.66
Assertiveness (E3)	0.69	0.56	0.56	0.55
Activity (E4)	0.66	0.64	0.65	0.65
Excitement Seeking (E5)	0.47	0.43	0.44	0.41
Positive Emotions (E6)	0.82	0.71	0.72	0.69
Openness to Fantasy (O1)	0.78	0.70	0.67	0.69
Openness to Aesthetics (O2)	0.86	0.81	0.79	0.80
Openness to Feelings (O3)	0.77	0.71	0.71	0.69
Openness to Actions (O4)	0.69	0.67	0.67	0.67
Openness to Ideas (O5)	0.76	0.60	0.60	0.58
Openness to Values (O6)	0.50	0.47	0.47	0.46
Trust (A1)	0.82	0.77	0.77	0.78
Straightforwardness (A2)	0.57	0.56	0.57	0.54
Altruism (A3)	0.66	0.59	0.61	0.55
Compliance (A4)	0.71	0.61	0.63	0.59
Modesty (A5)	0.72	0.58	0.54	0.56
Tender-Mindedness (A6)	0.64	0.60	0.59	0.59
Competence (C1)	0.58	0.48	0.46	0.48
Order (C2)	0.77	0.73	0.74	0.74
Dutifulness (C3)	0.59	0.57	0.58	0.58
Achievement Striving (C4)	0.79	0.76	0.77	0.77
Self-Discipline (C5)	0.80	0.76	0.76	0.74
Deliberation (C6)	0.73	0.61	0.63	0.58
Neuroticism	0.89	0.86	0.84	0.82
Extraversion	0.87	0.84	0.84	0.81
Openness	0.87	0.83	0.83	0.80
Agreeableness	0.83	0.80	0.80	0.74
Conscientiousness	0.89	0.86	0.86	0.82

Note. S5 = full (60-item) version of the "Short Five". XS5 = abbreviated (30-item) version of the "Short Five". Correlations between XS5 and NEO PI-R after partialling for (xs5part) or subtracting acquiescence are shown in the two rightmost columns.

Table J. Correlations with NEO PI-R: Spain

	s5	xs5	xs5p	xs5s
Anxiety (N1)	0.68	0.62	0.56	0.61
Angry Hostility (N2)	0.62	0.48	0.46	0.42
Depression (N3)	0.70	0.60	0.57	0.53
Self-Consciousness (N4)	0.62	0.50	0.51	0.44
Impulsiveness (N5)	0.60	0.53	0.50	0.53
Vulnerability (N6)	0.77	0.64	0.60	0.64
Warmth (E1)	0.66	0.58	0.57	0.56
Gregariousness (E2)	0.74	0.60	0.61	0.61
Assertiveness (E3)	0.61	0.42	0.43	0.40
Activity (E4)	0.60	0.57	0.56	0.56
Excitement Seeking (E5)	0.54	0.49	0.51	0.44
Positive Emotions (E6)	0.71	0.64	0.64	0.62
Openness to Fantasy (O1)	0.65	0.62	0.62	0.62
Openness to Aesthetics (O2)	0.73	0.71	0.70	0.70
Openness to Feelings (O3)	0.45	0.35	0.35	0.34
Openness to Actions (O4)	0.62	0.56	0.56	0.56
Openness to Ideas (O5)	0.64	0.39	0.38	0.39
Openness to Values (O6)	0.37	0.33	0.28	0.35
Trust (A1)	0.70	0.63	0.63	0.63
Straightforwardness (A2)	0.48	0.42	0.43	0.39
Altruism (A3)	0.43	0.32	0.33	0.29
Compliance (A4)	0.62	0.56	0.53	0.56
Modesty (A5)	0.58	0.46	0.46	0.46
Tender-Mindedness (A6)	0.33	0.24	0.24	0.23
Competence (C1)	0.51	0.48	0.48	0.46
Order (C2)	0.78	0.76	0.74	0.75
Dutifulness (C3)	0.48	0.41	0.40	0.38
Achievement Striving (C4)	0.69	0.61	0.61	0.57
Self-Discipline (C5)	0.71	0.55	0.56	0.53
Deliberation (C6)	0.74	0.70	0.69	0.68
Neuroticism	0.84	0.79	0.71	0.74
Extraversion	0.79	0.74	0.74	0.68
Openness	0.73	0.67	0.67	0.63
Agreeableness	0.64	0.58	0.58	0.52
Conscientiousness	0.80	0.77	0.78	0.68

Note. S5 = full (60-item) version of the “Short Five”. XS5 = abbreviated (30-item) version of the “Short Five”. Correlations between XS5 and NEO PI-R after partialling for (xs5part) or subtracting acquiescence are shown in the two rightmost columns.

Table K. Correlations with NEO PI-R: China

	s5	xs5	xs5p	xs5s
Anxiety (N1)	0.64	0.68	0.66	0.66
Angry Hostility (N2)	0.68	0.40	0.40	0.35
Depression (N3)	0.73	0.59	0.60	0.53
Self-Consciousness (N4)	0.64	0.44	0.49	0.37
Impulsiveness (N5)	0.59	0.53	0.46	0.50
Vulnerability (N6)	0.64	0.53	0.50	0.52
Warmth (E1)	0.65	0.59	0.58	0.57
Gregariousness (E2)	0.72	0.60	0.62	0.61
Assertiveness (E3)	0.69	0.54	0.52	0.53
Activity (E4)	0.55	0.54	0.55	0.54
Excitement Seeking (E5)	0.70	0.59	0.61	0.55
Positive Emotions (E6)	0.79	0.68	0.69	0.65
Openness to Fantasy (O1)	0.62	0.51	0.50	0.50
Openness to Aesthetics (O2)	0.71	0.71	0.70	0.69
Openness to Feelings (O3)	0.41	0.30	0.35	0.27
Openness to Actions (O4)	0.64	0.55	0.55	0.55
Openness to Ideas (O5)	0.70	0.51	0.53	0.48
Openness to Values (O6)	0.34	0.31	0.23	0.33
Trust (A1)	0.79	0.70	0.72	0.72
Straightforwardness (A2)	0.66	0.58	0.58	0.56
Altruism (A3)	0.67	0.57	0.57	0.54
Compliance (A4)	0.64	0.49	0.50	0.47
Modesty (A5)	0.65	0.54	0.54	0.54
Tender-Mindedness (A6)	0.58	0.47	0.43	0.42
Competence (C1)	0.44	0.39	0.39	0.38
Order (C2)	0.59	0.61	0.63	0.63
Dutifulness (C3)	0.49	0.44	0.46	0.47
Achievement Striving (C4)	0.55	0.54	0.55	0.54
Self-Discipline (C5)	0.63	0.58	0.53	0.57
Deliberation (C6)	0.65	0.60	0.56	0.59
Neuroticism	0.82	0.77	0.73	0.76
Extraversion	0.86	0.81	0.81	0.81
Openness	0.79	0.77	0.79	0.76
Agreeableness	0.80	0.76	0.76	0.75
Conscientiousness	0.75	0.73	0.72	0.73

Note. S5 = full (60-item) version of the “Short Five”. XS5 = abbreviated (30-item) version of the “Short Five”. Correlations between XS5 and NEO PI-R after partialling for (xs5part) or subtracting acquiescence are shown in the two rightmost columns.

Table L. Self-peer correlations: Estonia

	s5	xs5	xs5part	xs5subtr
Anxiety (N1)	0.44	0.38	0.38	0.37
Angry Hostility (N2)	0.45	0.41	0.41	0.41
Depression (N3)	0.48	0.38	0.39	0.40
Self-Consciousness (N4)	0.48	0.44	0.44	0.48
Impulsiveness (N5)	0.42	0.44	0.38	0.41
Vulnerability (N6)	0.43	0.40	0.39	0.38
Warmth (E1)	0.54	0.43	0.41	0.40
Gregariousness (E2)	0.54	0.52	0.48	0.50
Assertiveness (E3)	0.54	0.44	0.45	0.46
Activity (E4)	0.61	0.51	0.49	0.49
Excitement Seeking (E5)	0.52	0.46	0.47	0.47
Positive Emotions (E6)	0.59	0.56	0.57	0.57
Openness to Fantasy (O1)	0.37	0.37	0.37	0.37
Openness to Aesthetics (O2)	0.62	0.58	0.58	0.57
Openness to Feelings (O3)	0.49	0.43	0.44	0.47
Openness to Actions (O4)	0.44	0.43	0.39	0.40
Openness to Ideas (O5)	0.47	0.32	0.34	0.36
Openness to Values (O6)	0.28	0.18	0.21	0.22
Trust (A1)	0.38	0.30	0.31	0.30
Straightforwardness (A2)	0.45	0.46	0.42	0.50
Altruism (A3)	0.42	0.40	0.38	0.45
Compliance (A4)	0.49	0.44	0.44	0.47
Modesty (A5)	0.43	0.44	0.45	0.45
Tender-Mindedness (A6)	0.29	0.30	0.31	0.30
Competence (C1)	0.42	0.39	0.38	0.42
Order (C2)	0.51	0.54	0.55	0.55
Dutifulness (C3)	0.35	0.26	0.28	0.32
Achievement Striving (C4)	0.48	0.40	0.41	0.41
Self-Discipline (C5)	0.46	0.35	0.31	0.39
Deliberation (C6)	0.51	0.48	0.43	0.53
Neuroticism	0.50	0.47	0.45	0.47
Extraversion	0.67	0.64	0.63	0.64
Openness	0.60	0.56	0.56	0.59
Agreeableness	0.52	0.49	0.48	0.55
Conscientiousness	0.51	0.50	0.46	0.56

Note. S5 = full (60-item) version of the “Short Five”. XS5 = abbreviated (30-item) version of the “Short Five”. Correlations between XS5 and NEO PI-R after partialling for (xs5part) or subtracting acquiescence are shown in the two rightmost columns.

Table M. Internal consistencies (Cronbach alphas) in Finnish representative sample

	N	E	O	A	C
Raw scores	0.78	0.71	0.61	0.50	0.69
Partialing for acquiescence	0.79	0.73	0.65	0.53	0.73
Subtracting acquiescence	0.79	0.73	0.67	0.61	0.76

Table N. Principal component structure in the Finnish representative sample (rotated towards the US normative NEO PI-R structure using Procrustes rotation)

	N	E	O	A	C	Matrix
Anxiety (N1)	0.70	-0.11	0.00	-0.05	-0.13	0.98
Angry Hostility (N2)	0.67	-0.09	0.10	-0.31	-0.11	0.97
Depression (N3)	0.65	-0.21	0.13	-0.17	-0.17	0.95
Self-Consciousness (N4)	0.55	-0.44	-0.10	0.01	-0.09	0.91
Impulsiveness (N5)	0.36	0.21	-0.03	-0.16	-0.47	0.94
Vulnerability (N6)	0.67	-0.18	-0.10	0.07	-0.28	0.99
Warmth (E1)	-0.26	0.58	0.21	0.18	0.13	0.95
Gregariousness (E2)	-0.20	0.65	0.18	-0.02	0.07	0.96
Assertiveness (E3)	-0.19	0.37	0.34	-0.35	0.31	0.97
Activity (E4)	-0.33	0.61	-0.07	-0.04	0.29	0.77
Excitement Seeking (E5)	-0.13	0.25	0.34	-0.40	-0.17	0.79
Positive Emotions (E6)	-0.18	0.37	0.47	-0.11	0.11	0.74
Openness to Fantasy (O1)	0.05	0.38	0.30	0.14	-0.16	0.74
Openness to Aesthetics (O2)	0.09	0.30	0.33	0.35	-0.02	0.72
Openness to Feelings (O3)	0.26	0.22	0.43	0.15	0.32	0.89
Openness to Actions (O4)	-0.26	0.55	0.19	-0.02	0.00	0.68
Openness to Ideas (O5)	-0.03	-0.03	0.55	0.06	-0.06	0.91
Openness to Values (O6)	-0.08	0.14	0.66	0.10	-0.02	0.92
Trust (A1)	-0.23	0.31	0.11	0.52	-0.09	0.96
Straightforwardness (A2)	-0.04	0.04	0.17	0.47	0.33	0.84
Altruism (A3)	0.08	0.15	0.49	0.31	0.30	0.55
Compliance (A4)	-0.35	-0.13	0.26	0.43	0.17	0.78
Modesty (A5)	0.11	-0.41	-0.36	0.44	-0.12	0.85
Tender-Mindedness (A6)	0.15	0.27	0.18	0.54	0.13	0.96
Competence (C1)	-0.28	-0.50	0.23	0.21	0.60	0.65
Order (C2)	0.06	0.19	-0.37	0.15	0.51	0.88
Dutifulness (C3)	-0.01	0.18	-0.15	0.41	0.43	0.83
Achievement Striving (C4)	-0.27	0.44	-0.14	0.04	0.43	0.75
Self-Discipline (C5)	-0.20	0.17	-0.03	-0.03	0.65	0.98
Deliberation (C6)	-0.28	-0.50	0.23	0.21	0.60	0.93
Congruence	0.93	0.78	0.73	0.89	0.93	0.85
(cng)Part	0.92	0.82	0.90	0.91	0.95	0.90
(cng)Subtr	0.84	0.87	0.88	0.86	0.89	0.86

Table O. Principal component structure of XS5 in the Spanish sample (rotated towards the US normative NEO PI-R structure using Procrustes rotation)

	N	E	O	A	C	Matrix
Anxiety (N1)	0.66	-0.01	-0.13	0.08	-0.01	0.97
Angry Hostility (N2)	0.49	0.30	-0.19	-0.40	0.03	0.85
Depression (N3)	0.57	-0.16	0.05	0.13	-0.32	0.95
Self-Consciousness (N4)	0.37	-0.56	-0.06	-0.15	-0.03	0.71
Impulsiveness (N5)	0.47	0.25	0.07	-0.38	-0.32	0.96
Vulnerability (N6)	0.65	-0.02	-0.22	-0.04	-0.32	0.97
Warmth (E1)	-0.21	0.72	0.01	0.04	0.04	0.87
Gregariousness (E2)	-0.18	0.69	-0.03	0.11	0.12	0.97
Assertiveness (E3)	-0.15	0.53	0.04	-0.08	0.06	0.80
Activity (E4)	-0.17	0.29	0.24	-0.25	0.55	0.89
Excitement Seeking (E5)	-0.18	0.31	0.58	-0.21	0.06	0.63
Positive Emotions (E6)	-0.24	0.66	0.07	0.09	0.03	0.94
Openness to Fantasy (O1)	0.08	0.03	0.59	0.03	-0.09	0.89
Openness to Aesthetics (O2)	0.05	-0.02	0.59	0.00	0.00	0.95
Openness to Feelings (O3)	0.17	0.43	0.33	0.29	0.11	0.85
Openness to Actions (O4)	-0.12	0.31	0.59	-0.21	0.13	0.89
Openness to Ideas (O5)	0.00	-0.14	0.63	0.12	0.00	0.90
Openness to Values (O6)	-0.06	-0.03	0.44	-0.04	-0.02	0.94
Trust (A1)	-0.31	0.19	-0.10	0.35	-0.22	0.82
Straightforwardness (A2)	0.00	0.23	0.17	0.45	0.25	0.71
Altruism (A3)	0.11	0.30	0.01	0.64	0.11	0.91
Compliance (A4)	-0.50	-0.25	0.20	0.42	0.13	0.73
Modesty (A5)	0.29	-0.37	0.03	0.35	0.12	0.73
Tender-Mindedness (A6)	0.23	0.36	0.01	0.39	0.22	0.82
Competence (C1)	-0.44	0.24	0.11	-0.17	0.49	0.94
Order (C2)	0.30	-0.01	-0.09	-0.05	0.61	0.86
Dutifulness (C3)	0.17	0.22	0.12	0.14	0.62	0.78
Achievement Striving (C4)	-0.13	0.09	-0.09	-0.08	0.74	0.94
Self-Discipline (C5)	-0.24	-0.03	0.04	0.03	0.60	0.96
Deliberation (C6)	-0.31	-0.29	-0.13	0.40	0.27	0.86
Congruence	0.88	0.86	0.84	0.84	0.91	0.87

Table P. Principal component structure of the 60-item S5 in the Spanish sample (rotated towards the US normative NEO PI-R structure using Procrustes rotation)

	N	E	O	A	C	Matrix
Anxiety (N1)	0.66	0.08	-0.24	0.05	0.14	0.89
Angry Hostility (N2)	0.67	0.20	-0.11	-0.41	-0.01	0.94
Depression (N3)	0.67	-0.20	0.07	0.06	-0.27	0.98
Self-Consciousness (N4)	0.48	-0.59	-0.06	-0.05	-0.05	0.79
Impulsiveness (N5)	0.45	0.25	0.09	-0.32	-0.32	0.97
Vulnerability (N6)	0.73	-0.02	-0.24	0.03	-0.33	0.97
Warmth (E1)	-0.19	0.82	-0.05	0.15	0.06	0.90
Gregariousness (E2)	-0.26	0.64	-0.16	0.23	0.09	0.92
Assertiveness (E3)	-0.19	0.51	0.17	-0.27	0.10	0.93
Activity (E4)	-0.02	0.36	0.13	-0.33	0.39	0.97
Excitement Seeking (E5)	-0.16	0.31	0.62	-0.17	0.09	0.59
Positive Emotions (E6)	-0.40	0.67	0.04	0.09	0.06	0.87
Openness to Fantasy (O1)	0.09	0.08	0.63	0.11	-0.10	0.87
Openness to Aesthetics (O2)	0.07	-0.09	0.61	0.01	0.07	0.96
Openness to Feelings (O3)	0.21	0.42	0.41	0.18	0.06	0.94
Openness to Actions (O4)	-0.19	0.22	0.63	-0.11	0.18	0.93
Openness to Ideas (O5)	-0.09	-0.09	0.75	-0.05	0.10	0.99
Openness to Values (O6)	-0.06	0.14	0.43	0.01	0.04	0.90
Trust (A1)	-0.42	0.26	0.02	0.37	-0.12	0.92
Straightforwardness (A2)	0.10	0.28	0.12	0.48	0.36	0.70
Altruism (A3)	0.18	0.32	0.10	0.61	0.19	0.90
Compliance (A4)	-0.45	-0.15	0.13	0.52	-0.08	0.85
Modesty (A5)	0.35	-0.41	-0.03	0.33	0.00	0.75
Tender-Mindedness (A6)	0.33	0.28	0.06	0.53	0.13	0.88
Competence (C1)	-0.49	0.21	0.22	-0.16	0.53	0.95
Order (C2)	0.16	0.04	-0.12	0.09	0.64	0.95
Dutifulness (C3)	0.03	0.18	0.10	0.26	0.73	0.91
Achievement Striving (C4)	-0.07	0.12	0.08	-0.09	0.78	0.98
Self-Discipline (C5)	-0.12	0.00	-0.03	0.11	0.78	0.95
Deliberation (C6)	-0.33	-0.34	-0.07	0.33	0.37	0.94
Congruence	0.90	0.88	0.88	0.91	0.94	0.90

Table Q. Principal component structure of the XS5 in the Chinese sample (rotated towards the US normative NEO PI-R structure using Procrustes rotation)

	N	E	O	A	C	Matrix
Anxiety (N1)	0.75	-0.16	0.02	0.07	-0.10	0.97
Angry Hostility (N2)	0.53	0.27	0.14	-0.01	-0.40	0.63
Depression (N3)	0.60	-0.34	0.00	-0.15	-0.21	0.92
Self-Consciousness (N4)	0.45	-0.39	0.07	0.08	-0.20	0.87
Impulsiveness (N5)	0.45	0.33	-0.15	-0.18	-0.41	0.96
Vulnerability (N6)	0.69	0.01	-0.18	0.12	-0.31	0.97
Warmth (E1)	-0.23	0.66	0.18	-0.17	-0.04	0.72
Gregariousness (E2)	-0.24	0.73	0.04	-0.11	-0.02	0.97
Assertiveness (E3)	-0.19	0.30	0.42	-0.46	0.14	0.89
Activity (E4)	-0.12	0.66	0.18	-0.09	0.12	0.85
Excitement Seeking (E5)	-0.14	0.48	0.50	-0.08	-0.01	0.73
Positive Emotions (E6)	-0.18	0.51	0.26	0.21	-0.31	0.77
Openness to Fantasy (O1)	-0.13	0.48	0.31	-0.03	0.17	0.44
Openness to Aesthetics (O2)	0.13	0.14	0.49	0.01	0.35	0.88
Openness to Feelings (O3)	0.19	0.05	0.53	-0.26	0.10	0.77
Openness to Actions (O4)	-0.16	0.52	0.47	0.01	0.12	0.87
Openness to Ideas (O5)	0.04	-0.13	0.60	0.05	0.29	0.91
Openness to Values (O6)	-0.17	0.06	0.56	0.39	-0.21	0.77
Trust (A1)	0.02	0.47	-0.09	0.52	0.04	0.74
Straightforwardness (A2)	0.17	0.07	0.14	0.67	0.19	0.86
Altruism (A3)	-0.09	0.32	0.19	0.53	0.09	0.90
Compliance (A4)	-0.33	-0.19	0.25	0.24	-0.33	0.52
Modesty (A5)	0.06	-0.47	-0.25	0.49	-0.11	0.85
Tender-Mindedness (A6)	0.26	0.22	0.05	0.45	0.27	0.82
Competence (C1)	-0.55	0.22	0.12	-0.07	0.47	0.95
Order (C2)	0.08	-0.13	-0.18	-0.10	0.54	0.92
Dutifulness (C3)	0.13	0.13	-0.14	0.29	0.57	0.84
Achievement Striving (C4)	-0.18	-0.06	0.14	0.01	0.61	0.90
Self-Discipline (C5)	-0.37	-0.05	0.11	0.25	0.41	0.82
Deliberation (C6)	-0.33	-0.46	0.27	0.13	0.26	0.75
Congruence	0.89	0.84	0.83	0.75	0.81	0.82

Table R. Principal component structure of the 60-item S5 in the Chinese sample (rotated towards the US normative NEO PI-R structure using Procrustes rotation)

	N	E	O	A	C	Matrix
Anxiety (N1)	0.70	-0.08	-0.04	-0.06	-0.13	0.99
Angry Hostility (N2)	0.74	0.10	-0.03	-0.27	-0.20	0.93
Depression (N3)	0.74	-0.28	-0.05	-0.07	-0.20	0.97
Self-Consciousness (N4)	0.59	-0.45	-0.03	0.08	-0.21	0.92
Impulsiveness (N5)	0.39	0.33	0.04	-0.14	-0.54	0.94
Vulnerability (N6)	0.70	-0.02	-0.16	0.11	-0.40	0.98
Warmth (E1)	-0.28	0.78	0.07	0.02	0.11	0.87
Gregariousness (E2)	-0.21	0.76	0.06	0.00	-0.10	0.99
Assertiveness (E3)	-0.34	0.45	0.22	-0.40	0.39	1.00
Activity (E4)	0.05	0.77	0.13	-0.19	-0.04	0.78
Excitement Seeking (E5)	-0.19	0.51	0.60	-0.12	0.09	0.69
Positive Emotions (E6)	-0.29	0.69	0.11	0.18	-0.07	0.91
Openness to Fantasy (O1)	0.06	0.21	0.60	0.01	-0.11	0.92
Openness to Aesthetics (O2)	0.13	0.02	0.67	0.13	0.20	0.99
Openness to Feelings (O3)	0.36	0.31	0.31	-0.10	0.32	0.91
Openness to Actions (O4)	-0.26	0.53	0.57	-0.14	0.09	0.90
Openness to Ideas (O5)	-0.14	-0.12	0.76	-0.03	0.22	0.99
Openness to Values (O6)	-0.22	0.14	0.40	0.36	-0.22	0.71
Trust (A1)	0.04	0.42	-0.01	0.63	0.16	0.78
Straightforwardness (A2)	0.15	0.13	0.03	0.68	0.33	0.88
Altruism (A3)	-0.06	0.46	0.24	0.55	0.13	0.91
Compliance (A4)	-0.40	-0.08	0.13	0.37	-0.18	0.75
Modesty (A5)	0.06	-0.44	-0.08	0.57	-0.26	0.85
Tender-Mindedness (A6)	0.30	0.15	0.12	0.55	-0.01	0.91
Competence (C1)	-0.58	0.17	0.15	-0.13	0.54	0.95
Order (C2)	0.03	-0.11	-0.21	0.12	0.65	0.95
Dutifulness (C3)	0.05	0.03	-0.08	0.40	0.61	0.92
Achievement Striving (C4)	-0.22	-0.06	0.38	-0.14	0.58	0.85
Self-Discipline (C5)	-0.44	-0.13	0.04	0.22	0.41	0.80
Deliberation (C6)	-0.31	-0.31	0.09	0.04	0.48	0.94
Congruence	0.92	0.90	0.89	0.87	0.90	0.90

Table S. Fit statistics for the ESEM models (XS5) in each country

Country	χ^2	df	p	CFI	TLI	RMSEA	SRMR
China	560.851	295	< 0.0001	0.797	0.701	0.069	0.046
Estonia	664.763	295	< 0.0001	0.883	0.827	0.056	0.037
UK	464.519	295	< 0.0001	0.769	0.659	0.077	0.056
Spain	493.121	295	< 0.0001	0.865	0.801	0.051	0.042
Finland	939.983	295	< 0.0001	0.875	0.816	0.057	0.035
Germany	617.344	295	< 0.0001	0.793	0.695	0.071	0.048

Table T. Fit statistics for the ESEM models (NEO PI-R) in each country

Country	χ^2	df	p	CFI	TLI	RMSEA	SRMR
China	584.956	295	0	0.885	0.830	0.072	0.036
Estonia	1093.384	295	0	0.854	0.784	0.083	0.037
UK	620.737	295	0	0.775	0.668	0.106	0.052
Spain	578.069	295	0	0.905	0.861	0.061	0.038
Finland	600.756	295	0	0.712	0.575	0.108	0.057
Germany	655.857	295	0	0.874	0.815	0.075	0.039
